# Mechanisms for the Evolution of a Derived Function in the Ancestral Glucocorticoid Receptor

**DOI:** 10.1371/journal.pgen.1002117

**Published:** 2011-06-16

**Authors:** Sean Michael Carroll, Eric A. Ortlund, Joseph W. Thornton

**Affiliations:** 1 Department of Organismic and Evolutionary Biology, Harvard University, Cambridge, Massachusetts, United States of America; 2 Department of Biochemistry, Emory University School of Medicine, Atlanta, Georgia, United States of America; 3 Howard Hughes Medical Institute, Center for Ecology and Evolutionary Biology, University of Oregon, Eugene, Oregon, United States of America; University of Michigan, United States of America

## Abstract

Understanding the genetic, structural, and biophysical mechanisms that caused protein functions to evolve is a central goal of molecular evolutionary studies. Ancestral sequence reconstruction (ASR) offers an experimental approach to these questions. Here we use ASR to shed light on the earliest functions and evolution of the glucocorticoid receptor (GR), a steroid-activated transcription factor that plays a key role in the regulation of vertebrate physiology. Prior work showed that GR and its paralog, the mineralocorticoid receptor (MR), duplicated from a common ancestor roughly 450 million years ago; the ancestral functions were largely conserved in the MR lineage, but the functions of GRs—reduced sensitivity to all hormones and increased selectivity for glucocorticoids—are derived. Although the mechanisms for the evolution of glucocorticoid specificity have been identified, how reduced sensitivity evolved has not yet been studied. Here we report on the reconstruction of the deepest ancestor in the GR lineage (AncGR1) and demonstrate that GR's reduced sensitivity evolved before the acquisition of restricted hormone specificity, shortly after the GR–MR split. Using site-directed mutagenesis, X-ray crystallography, and computational analyses of protein stability to recapitulate and determine the effects of historical mutations, we show that AncGR1's reduced ligand sensitivity evolved primarily due to three key substitutions. Two large-effect mutations weakened hydrogen bonds and van der Waals interactions within the ancestral protein, reducing its stability. The degenerative effect of these two mutations is extremely strong, but a third permissive substitution, which has no apparent effect on function in the ancestral background and is likely to have occurred first, buffered the effects of the destabilizing mutations. Taken together, our results highlight the potentially creative role of substitutions that partially degrade protein structure and function and reinforce the importance of permissive mutations in protein evolution.

## Author Summary

A central question in molecular evolution is how changes in the genetic, structural, and biophysical properties of proteins generate new functions. Ancestral sequence reconstruction (ASR) allows long-extinct proteins to be resurrected and characterized in the laboratory and allows the mechanisms for evolutionary shifts in protein functions to be studied experimentally. We used ASR to study the earliest functions and evolution of the glucocorticoid receptor (GR), a hormone-activated transcription factor and critical regulator of vertebrate physiology. We reconstructed the first GR ancestor and showed that this ancient gene evolved dramatically reduced sensitivity to hormone shortly after its birth by gene duplication some 450 million years ago, a function that persists in GRs to this day. Using site-directed mutagenesis, X-ray crystallography, and computational predictions of protein stability, we found that the shift to reduced GR sensitivity was driven by two large-effect mutations that destabilized the receptor-hormone complex. The combined effect of these mutations is so strong that a third mutation, apparently neutral in the ancestral background, evolved to buffer their degenerative effects. Our results suggest a creative role for mutations that partially degrade protein form and function and highlight the importance of interactions between mutations in evolutionary processes and protein functions.

## Introduction

A central goal in studies of molecular evolution is to reveal the genetic, structural, and biophysical mechanisms by which protein functions have evolved [1]–[6]. Ancient proteins and DNA are seldom directly available, but the traces of their evolutionary history are found in their extant descendants [7]. Direct comparisons among present-day proteins can sometime yield insights into the sequence and structural mechanisms that underlie functional differences [8]–[11]. Such “horizontal” comparisons, however, cannot determine which protein features are ancestral and which are derived, so they are not suited to reconstructing the events that produced functional diversity [12]. Further, because the effect of a mutation on protein structure and function often depends on the residues present at other sequence sites [13]–[17], studies of extant proteins may often be unsuited to revealing the effects of mutations in the historical backgrounds in which they occurred [12].

Ancestral sequence reconstruction (ASR) allows the forms and functions of ancient proteins to be studied experimentally. Beginning with an alignment of extant sequences, the maximum likelihood phylogeny and best-fit probabilistic model of evolution are inferred; the most likely ancestral sequence at any node – defined as the sequence with the highest probability of delivering all the observed extant sequences – can then be identified [18]. These ancestral protein sequences can be “resurrected” using gene synthesis and cell culture or *in vitro* expression systems and then characterized using the same methods typically applied to study extant proteins. This approach allows hypotheses about the ancestral and derived characteristics of proteins to be tested experimentally. It also allows the historical interval during which structure and function changed to be identified and the causal role of specific historical mutations in the ancestral background to be determined.

The glucocorticoid and mineralocorticoid receptors (GR and MR) are paralogous hormone-regulated transcription factors that have served as useful models for studying protein evolution [15], [16], [19]. GR and MR have a modular domain structure that includes a well-conserved DNA-binding domain (DBD) and a moderately conserved ligand-binding domain (LBD) – which binds the hormone, changes conformation, and attracts coactivator proteins that potentiate transcription of nearby target genes; they also contain poorly conserved hinge and N-terminal domains. In most bony vertebrates, the intrinsic functions of the GR and MR LBDs differ in both specificity and sensitivity. GR is more specific, being activated by high doses of the adrenal hormone cortisol to regulate aspects of immunity, glucose metabolism, and the long-term stress response [20], [21]. MR, in contrast, is activated by the adrenal mineralocorticoids aldosterone or deoxycorticosterone, as well as cortisol (albeit with somewhat lower sensitivity), and primarily regulates osmotic homeostasis. GR is also considerably less sensitive than MR, often requiring concentrations several orders of magnitude higher for activation [19], [22], [23].

**Figure 1:**
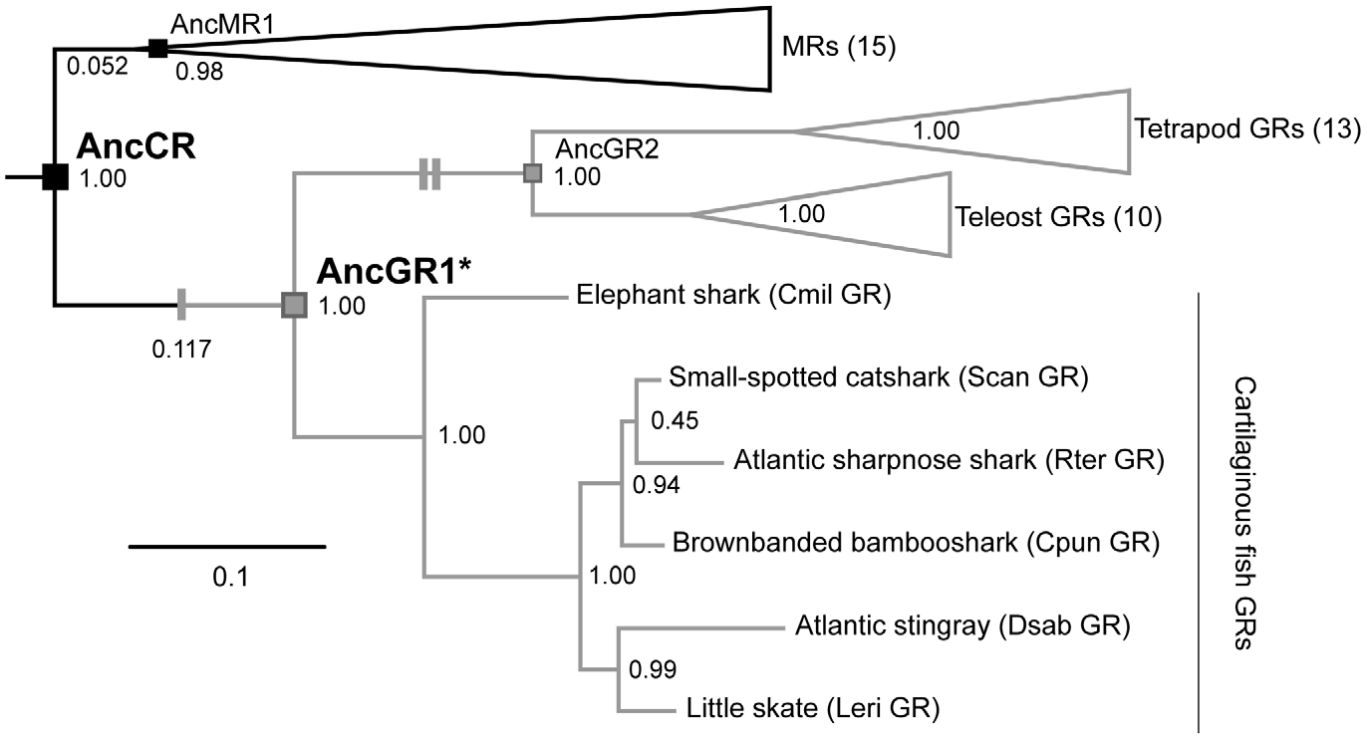
Simplified phylogeny of corticosteroid receptors. Ancestral sequences are shown at relevant nodes: AncCR, the last common ancestor of all MRs and GRs; AncGR1, the GR ancestor of cartilaginous fishes and bony vertebrates; AncGR2, the GR ancestor of ray- and lobe-finned fishes (including tetrapods); AncMR1, the MR ancestor of cartilaginous fishes and bony vertebrates. (AncGR1.0 and AncGR1.1 are different reconstructions of node AncGR1, inferred from datasets with different taxon sampling.) Black, high sensitivity receptors; gray, low sensitivity receptors. Single and double gray dashes mark functional shifts towards reduced sensitivity and increased specificity, respectively. Support values are the chi-square statistic (1 – p, where p equals the estimated probability that a node could occur by chance alone) calculated from approximate likelihood ratios. The length of branches from AncCR to AncMR1 and to AncGR1, expressed as the mean number of substitutions per site, are indicated in parentheses.

Some information is available on GR and MR evolution. The two paralogs descend by duplication from a single ancestral corticosteroid receptor (AncCR), which existed in an ancient jawed vertebrate ∼450 million years ago, before the divergence of bony vertebrates from cartilaginous fishes (Figure 1) [19], [24]. Reconstruction and experimental analysis showed that AncCR, like the extant MRs, was extremely sensitive to both mineralocorticoids and glucocorticoids, and its structure was MR-like, as well [19]. Subsequent work revealed that GR's specificity for glucocorticoids evolved later in the lineage leading to bony vertebrates, after the divergence of cartilaginous fishes but before the split of ray-finned fish from the lineage leading to tetrapods and lobe-finned fish, due to a small specific set of historical mutations [15], [16], [19] (Figure 1).

The evolutionary causes of GR's reduced hormone sensitivity are not known. In the little skate – the only cartilaginous fish studied to date – GR is a low-sensitivity, broad-spectrum receptor: like MR, it responds to both glucocorticoids and mineralocorticoids, but it is unique in requiring high concentrations of either type of hormone to activate it. The difference in receptor sensitivity between the GR and MR is thought to have physiological consequences: in several elasmobranch species, the same corticosteroids appear to regulate both stress and osmolarity [25]–[28], and the highest titres are associated with stress conditions [29], [30]. These observations suggest that GR regulates stress in response to high doses of hormones, while MR regulates osmolarity in response to much lower doses [23].

Based on these data, we hypothesize that GRs' reduced sensitivity to all hormones was an independent evolutionary event that occurred before cartilaginous fishes split from bony vertebrates, and before glucocorticoid specificity evolved in the GRs of bony vertebrates [15], [19]. Here we report on experiments to test this hypothesis and determine the genetic, structural, and biophysical mechanisms by which GR's reduced hormone sensitivity evolved. We first resurrected the LBD of AncGR1 (Figure 1) – the GR protein present in the common ancestor of bony and cartilaginous vertebrates and the earliest node after the GR-MR split – and then used functional assays, X-ray crystallography, site-directed mutagenesis, and computational predictions of biophysical parameters to dissect the mechanisms by which GR evolved. We show that after its initial birth by gene duplication, a small number of mutations that partially degraded its structure, stability, and function caused GR to become a novel low-sensitivity receptor.

## Results

### Isolation and Characterization of Cartilaginous Fish GRs

Statistical confidence in ASR depends in part on taxon sampling in groups descending directly from the node of interest [31]–[33]. Although GR sequences are available from many bony vertebrates, only a single GR sequence from cartilaginous fishes has been previously sequenced. We therefore isolated additional GRs sampled from throughout the cartilaginous fishes and characterized the functions of their LBDs. Specifically, we isolated GRs from four elasmobranch species – the Atlantic sharpnose shark (*Rhizoprionodon terraenovae*), brownbanded bambooshark (*Chiloscyllium punctatum*), small-spotted catshark (*Scyliorhinus canicula*), and the Atlantic stingray (*Dasyatis sabina*) – and one holocephalan, the elephant shark (*Callorhincus milii*) (Figure 1).

**Figure 2:**
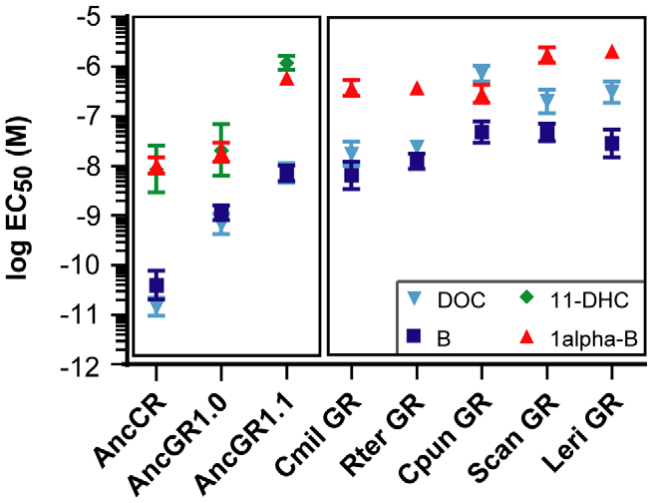
AncGR1 and its descendents evolved reduced hormone sensitivity. Ligand-dependent transcriptional activation of receptors was measured in the presence of increasing concentrations of hormone using a luciferase reporter gene assay. Dose-response curves were calculated for receptor-hormone pairs and plotted as the log effective concentration of hormone for half maximal activation (log EC_50_) in molar with standard error. Larger values, lower sensitivity; smaller values, higher sensitivity. Hormones are: 11-deoxycorticosterone (DOC), corticosterone (B), 11-dehydrocorticosterone (11-DHC), and 1α-hydroxycorticosterone (1α-B). Cartilaginous fish species are shown in (Figure 1). 11-DHC did not activate cartilaginous fish GRs in our assay (defined as <2-fold activation for EC_50_>1 µM of hormone) and is not shown for these receptors.

We used a luciferase reporter gene expression assay to characterize the sensitivity of each LBD to four major corticosteroids present in elasmobranchs – 11-deoxycorticosterone (DOC), corticosterone, 1alpha-hydroxycorticosterone, and 11-dehydrocorticosterone [34], [35]. All elasmobranch GRs were low-sensitivity receptors activated by multiple corticosteroids, except for the *D. Sabina* GR, which did not activate transcription in the presence of any hormone. All hormone-activated cartilaginous fish GRs were most sensitive to DOC and corticosterone. The receptors had EC_50_ values (the hormone concentration required to elicit half-maximal activation) for these steroids in the 10^−8^ to 10^−6^ M range (Figure 2, Table S1), typical of the EC_50_s of bony fish GRs for glucocorticoids but two to four orders of magnitude greater than AncCR or the MRs of bony vertebrates [19]. These observations are consistent with a model that after duplication of AncCR – which was highly sensitive to a broad array of corticoisteroids – GR evolved reduced sensitivity without a shift in specificity, explaining the observed characteristics of AncGR1 and the GRs of extant elasmobranchs; later – after elasmobranchs diverged from bony vertebrates – the narrower specificity for glucocorticoids that characterizes the GRs of present-day tetrapods and teleosts evolved (Figure 1).

### Reconstruction and Functional Analysis of the Ancestral GR

The new cartilaginous fish GR sequences were added to a dataset of 97 other steroid receptor sequences and aligned for phylogenetic analyses and ancestral sequence reconstruction. The maximum likelihood phylogeny was generally well supported and in agreement with previously published trees [19], except for the placement of the agnathan receptors (Figure S1, Table S2).

The hypothesis that GR's functions are derived can be tested experimentally and by sequence analysis of evolutionary rates. This hypothesis predicts that the rate of amino acid evolution after duplication of AncCR should be faster in the lineage leading to the GRs than in that leading to the MRs, which retain the ancestral functions [3]. Branch lengths between two nodes represent the mean probability of substitution per site, which equals the product of evolutionary rate times time. The branch leading from AncCR to AncGR1 and the branch leading from AncCR to AncMR1 (MR in the same ancestral species—the common ancestor of jawed vertebrates) cover exactly the same period of time, so any authentic differences in length must be due to differences in evolutionary rate. As predicted, there are 36 differences between the AncCR and AncGR1.1, compared to 16 between AncCR and AncMR1, and the estimated amino acid replacement rate 2.25 times greater on the GR branch than on the MR branch (Figure 1), but this difference did not reach formal statistical significance (p = 0.09) using a likelihood ratio test.

To more decisively test the hypothesis that GR's functions changed between AncCR and AncGR1, we used ancestral reconstruction. We inferred the sequence of AncGR1 assuming the best-fit model and integrating over plausible phylogenies weighted by their posterior probabilities [36]. The denser taxon sampling of this study was found to improve confidence in the inferred AncGR1 sequence compared to the previously published version, which was inferred from an alignment that included only a single cartilaginous fish [15]. The updated reconstruction, which we named AncGR1.1, differs at 7% of sites from the original reconstruction (AncGR1.0), with a higher mean posterior probability across all sites (0.951 vs. 0.930), a greater number of sites reconstructed with 100% posterior probability, and fewer sites reconstructed with plausible alternate states (Figure S2, Table S3). Other previously reconstructed ancestral steroid receptor sequences, such as AncCR and AncGR2 (the GR gene in the last common ancestor of ray- and lobe-finned fishes, including tetrapods) [19], were affected to a much lesser extent by including additional cartilaginous fish sequences.

We then characterized the functions of the AncGR1.1 LBD by synthesizing a nucleic acid sequence that codes for it, subcloning that sequence into an expression construct, and assaying its sensitivity to the same suite of corticosteroids using the luciferase reporter assay. As predicted, we found that AncGR1.1 is activated by the same broad suite of hormones as AncCR, but markedly higher doses are required. For all ligands tested – including the classic mineralocorticoid DOC – AncGR1.1 was 25- to 530-fold less responsive to hormone than AncCR (Figure 2, Table S1). These data allow us to trace on the phylogeny two separate shifts in the evolution of GRs from a high-sensitivity, promiscuous corticosteroid receptor: first, the evolution of reduced hormone sensitivity, and later a loss of sensitivity to mineralocorticoids (Figure 1).

To determine whether AncGR1.1′s reduced sensitivity could be an artifact of uncertainty in the ancestral sequence reconstruction, we introduced plausible alternate states into the maximum likelihood ancestral sequence and repeated the experimental characterization. None of these contradicted the finding that AncGR1.1 has markedly reduced hormone sensitivity compared to AncCR (Table S1). Taken together, these observations indicate that reduced sensitivity evolved in the GR lineage after duplication of AncCR but before the split of cartilaginous from bony vertebrates, and this conclusion is robust to uncertainty about the ancestral reconstruction.

### Genetic Basis of Reduced AncGR1.1 Sensitivity

We next sought to identify the genetic mechanisms that caused reduced hormone sensitivity to evolve. Because the shift in function occurred on the branch between AncCR and AncGR1.1, the initial set of candidate mutations includes the 36 historical substitutions that occurred on this same branch. At 17 of these sites, the same derived state is present in both AncGR1.1 and AncGR1.0: AncGR1.0 is much more similar in sensitivity to AncCR than AncGR1.1 is (Figure 2), so substitutions at these sites are unlikely to represent the major-effect mutations. Of the 19 substitutions that are unique to AncGR1.1, twelve represent biochemically conservative replacements (e.g., D/E, I/L, K/R, S/T). Only one of the others is in a position predicted to contact ligand based on the crystal structures of other steroid receptors [15], [37]; this substitution (A36G) was previously tested in AncCR and found to have no significant effect on sensitivity to DOC or other corticosteroids [19]. We therefore prioritized the six remaining biochemically radical replacements as the best candidates for having caused the evolution of reduced sensitivity.

**Figure 3:**
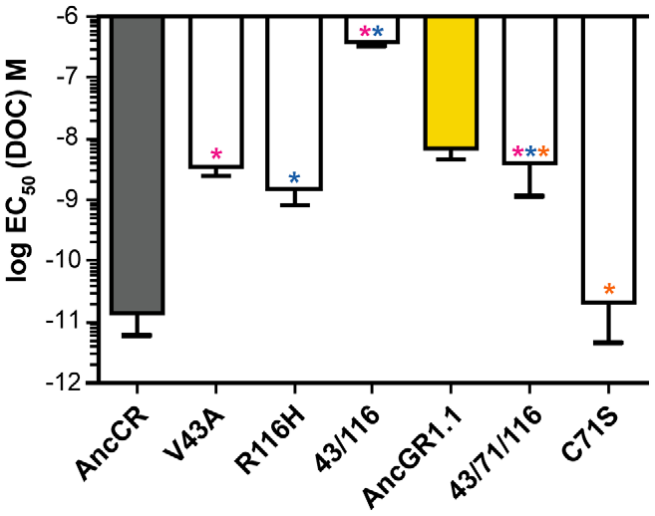
Three historical substitutions in AncCR recapitulate the evolution of reduced sensitivity. Shown is the log EC_50_ of molar DOC with the highly sensitive ancestor (AncCR, gray), the low sensitivity descendent (AncGR1.1, yellow), and AncCR mutants. Single and combination mutants are denoted with colored asterisks: V43A, pink; R116H, blue; C71S, orange.

We introduced each candidate mutation into the maximum likelihood (ML) AncCR background using site-directed mutagenesis and tested its effect on hormone sensitivity in the luciferase reporter gene assay with increasing concentrations of DOC. Two substitutions – V43A and R116H – markedly reduced AncCR's sensitivity to hormone, increasing the receptor's EC_50_ of DOC by at least two orders of magnitude to AncGR1.1-like values (Figure 3, Table 1). The others had much weaker effects on sensitivity. The double mutant V43A/R116H was severely compromised, with an EC_50_ for DOC ∼10,000 times greater than AncCR and more than 50 times greater than even AncGR1.1. These results indicate that V43A and R116H are large-effect historical mutations that are more than sufficient to recapitulate the evolution of the low-sensitivity AncGR1.1. They also indicate that the effects of these two mutations on receptor sensitivity must have been partially buffered by additional substitutions that occurred during the same interval.

**Table 1:** Response of ancestral and mutant receptors to 11-deoxycorticosterone (DOC).

Receptor^a^	log EC_50_ b	SE	Fold change in EC_50_ c
AncCR	−10.870	0.34	-
AncGR1.1	−8.144	0.20	532.1
C71S	−10.690	0.64	1.5
A107S	−10.350	0.24	3.3
K83Q	−10.330	0.41	3.5
L1M	−10.250	0.30	4.2
Q211E	−10.170	0.43	5.0
Q113K	−9.773	0.43	12.5
R116H	−8.812	0.28	114.3
V43A	−8.443	0.15	267.3
43/71/116	−8.387	0.54	304.1
43/116	−6.418	0.06	28313.9

a All mutations were introduced into the AncCR background; AncGR1.1 is shown for comparison. Mutations are listed in order of the magnitude of their effect.

b The log effective concentration of hormone for half-maximal activation plus standard error, calculated from nonlinear regression of triplicate reactions in a reporter gene assay.

c Fold change in EC_50_ indicates the ratio of EC_50_ of the listed receptor to the EC_50_ of AncCR, both for deoxycorticosterone. Positive values indicate reduced sensitivity.

### Crystal Structure of the AncGR1:DOC Complex

To understand the structural basis of reduced GR sensitivity and identify other important substitutions, we purified AncGR1.1 expressed in *E. coli* and used X-ray crystallography to determine its atomic structure in complex with DOC at 1.95 Å resolution (see Table S4). AncGR1.1 adopts the classic steroid receptor active conformation [38], consisting of three helical layers, an internal ligand cavity bounded by helices 3, 5, 6, 7, and 10, well-defined ligand density within the cavity, and a surface for coactivator binding formed by helices H3, H5, and H12 [39].

**Figure 4:**
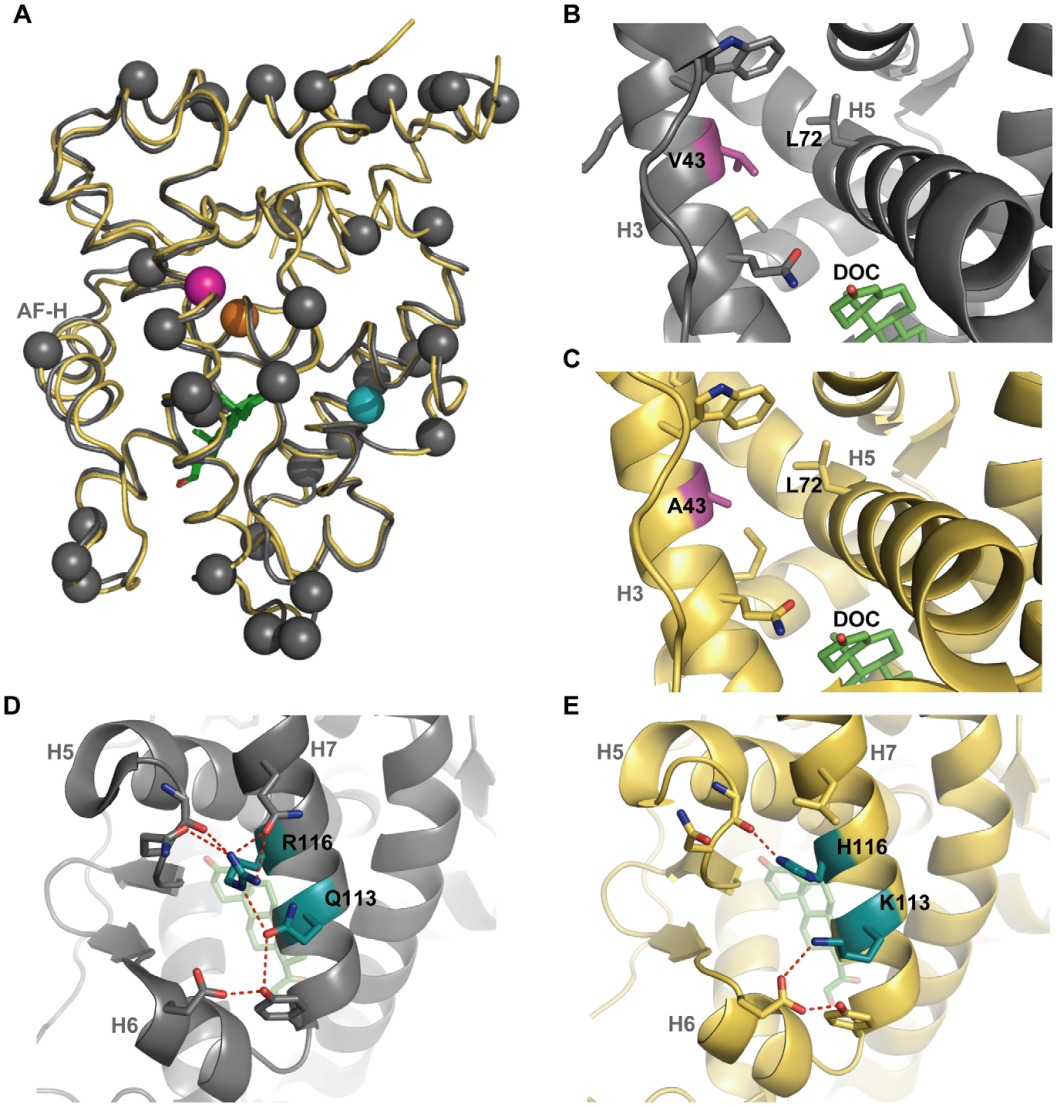
The crystal structure of AncGR1.1-LBD in complex with DOC (yellow, PDB 3RY9) compared to the previously solved structure of AncCR with DOC (gray, PDB 2Q3Y). Green sticks indicate DOC; possible hydrogen bonding indicated by red dashes. Helices are indicated by light grey text; AF-H equals H12, the activation function helix. A) AncCR and AncGR1.1 are highly structurally conserved despite 36 substitutions between them. Spheres show sequence differences: pink, position 43; blue, position 116; orange, position 71. B) and C) show the structural environment around position 43. The substitution of V43 in AncCR (B) in place of A43 in AncGR1.1 (C) is thought to weaken the hydrophobic interactions of the surrounding helices. D) and E) show the structural neighborhood of position 116. Replacing R116 (D) with H116 (E), along with a Q113K substitution, significantly reduces and rearranges ancestral hydrogen bonding.

The conformation of AncGR1.1 is very similar to the previously determined AncCR crystal structure, with a root mean square deviation (RMSD) in backbone atom position of only 0.66 Å (Figure 4A), and most of the larger deviations are far from the ligand. The side chain identity of all residues within 4 Å of DOC are conserved except for A36G, which alters a ligand-contacting residue but has no discernible effect on hormone sensitivity [19]. These results indicate that AncGR1.1′s reduced sensitivity to hormone must be due to indirect mechanisms not involving contacts with the ligand, such as changes to intraprotein contacts that affect the stability of the protein-hormone complex.

### Desensitizing Mutations Caused a Loss of Intra-Protein Interactions

To understand the mechanisms by which mutations V43A and R116H reduced hormone sensitivity, we first examined the apparent roles of these residues in the AncCR and AncGR1.1 crystal structures. Position 43 faces inward on the middle of H3, just above the ligand where H3 packs against H5 and forms part of the coactivator-binding cleft. In AncCR, Val43 packs tightly against neighboring hydrophobic residues, making van der Waals contacts with Leu72, presumably stabilizing H3 and H5, which participate in forming both the coactivator interface and the ligand pocket (Figure 4B). In AncGR1.1, the smaller side chain of Ala43 loses its van der Waals contacts to Leu72, opening a small cavity in this region (Figure 4C). The poor packing that results is expected to destabilize the receptor-ligand complex.

Position 116 is situated on H7, the opposite side of the protein from site 43. In AncCR, R116 is a hub in a network of hydrogen bonds between H7 and residues in H5 and H6 (Figure 4D). In AncGR1.1, this hydrogen-bond network is much sparser, largely due to the replacement of Arg116 with His (Figure 4E). The loss of favorable interactions presumably destabilizes these helices, the ligand pocket, and possibly the coactivator interface.

We also noted that a third historical substitution in this region, Q113K, abolishes other hydrogen bonds in the same network as Arg116H. When we introduced Q113K into AncCR, it also reduced sensitivity, though its effect was considerably smaller than those of V43A and R116H (Table 1). The loss of favorable interactions in the atomic structure due to substitutions at sites 43, 116 and 113, together with the experimental finding that these mutations recapitulate the evolutionary decline in AncCR's hormone sensitivity, suggests that AncGR1's novel function – its reduced sensitivity to corticosteroids – evolved because of the partial degeneration of ancestral structures and functions.

### C71S Buffers against Desensitizing Mutations

Because introducing mutations V43 and R116H together into AncCR reduces sensitivity to an extent greater than the historical difference between AncCR and AncGR1, other historical substitutions during the same interval must have buffered the impact of these large-effect mutations. Of the remaining candidate mutations, one – C71S – occurred at a site already known to have a strong positive effect on receptor function in extant steroid hormone receptors: introducing serine at the homologous site in mammalian GRs (F602S) dramatically improves bacterial expression, solubility, and crystallization [15], [16], [37], [40]–[2]. To test the hypothesis that the historical acquisition of Ser71 buffered the effect of mutations at sites 43 and 116, we introduced mutation C71S into AncCR-V43A/R116H background. As predicted, this additional change improved sensitivity by ∼90-fold, yielding a receptor with DOC sensitivity similar to that of AncGR1.1. In isolation, however, the C71S substitution has no discernable effect on AncCR sensitivity (Figure 3, Table 1).

**Figure 5:**
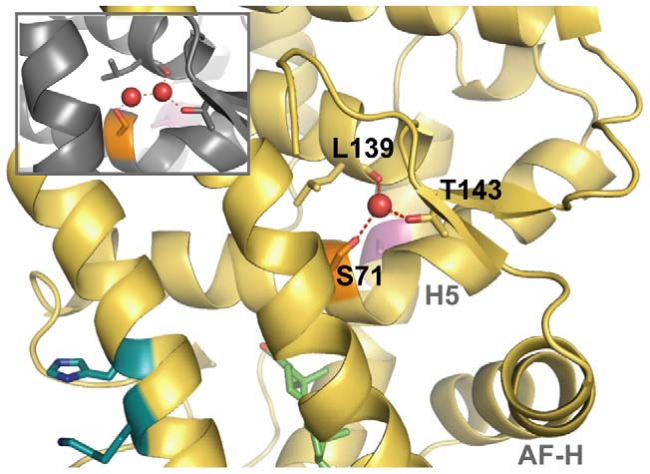
The structural context of site 71. S71 (orange) in AncGR1.1 (Chain B, yellow) forms a water-mediated hydrogen bond with the carbonyl carbon of L139 and the T143 side chain. In AncCR, the side chain of an engineered S71 mutant adopts a slightly different conformation (*inset*). The C71S substitution increases the hydrophilicity of the region. Also shown are the relative locations of the ligand, DOC (green); V43A (pink); the R116H and Q113K substitutions (blue); possible hydrogen bonding (red dashes); and relevant helices (light grey text).

The biophysical mechanism for this buffering effect is not clear. All previously crystallized corticosteroid receptors, ancestral and extant, have had Ser71 engineered into them to aid in protein expression and crystallization [15], [16], [37], [40]–[2]; comparison to receptor agonist structures lacking Ser71 is therefore not possible. In both the AncCR and AncGR1.1 structures, this site is located on H 5 in the central core of the protein, just above the ligand-binding pocket, bordering a distinctive kink in H5 (Figure 5). Ser71 is adjacent to a highly solvated channel next to the hydrophobic core of the receptor, and a serine substitution would increase the hydrophilicity of the region compared to the ancestral cysteine. It appears that Ser71 in Chain B of AncGR1.1 might stabilize the receptor through direct and water-mediated hydrogen bonds that a cysteine would not form (Figure 5). This is not a strictly conserved mechanism, however, because in the structures of AncCR-C71S (Figure 5, *inset*) and Chain A of AncGR1.1, the polar Ser71 side chain occupies an alternate conformation: it interacts with water molecules in the channel, but the bond network varies among the structures. An alternate explanation for the buffering effect of C71S is that it may facilitate proper folding and solubility of the protein, an effect that could have a more beneficial effect on receptors with less stable native conformations, such as those carrying the V43A or R116H substitutions.

### Desensitizing Mutations Are Predicted to Be Thermodynamically Destabilizing

**Figure 6:**
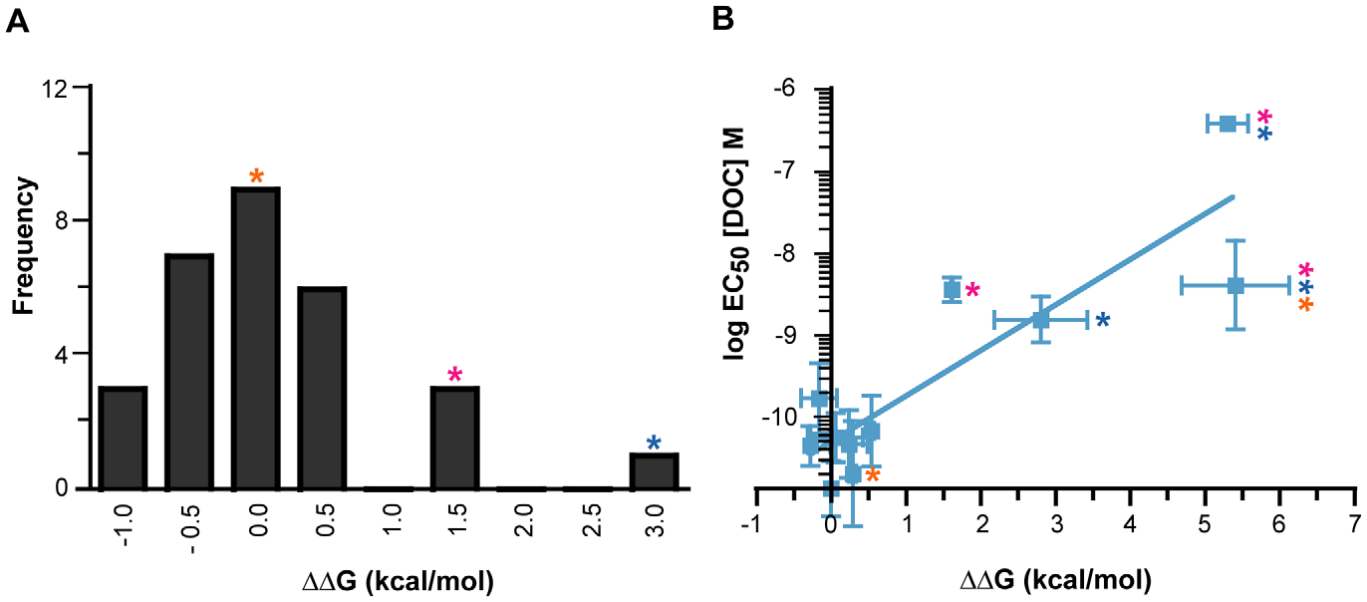
The effect of historical GR substitutions on AncCR protein stability, predicted by FoldX [**43**]. A) The distribution of stability effects (ΔΔG) for almost all (n = 29 of 36) single GR substitutions in the AncCR background. Colored asterisks indicate bins with notable mutants: V43A (pink), R116H (blue), and C71S (orange). B) For those mutants in which hormone sensitivity was assessed (n = 10, plus AncCR), the predicted loss of stability correlates well with the observed loss of sensitivity towards DOC. Linear regression with C71S mutants, r^2^ = 0.78; without, r^2^ = 0.89. Single and combination mutants are colored as in (A). X values are the average of five FoldX runs with standard deviation; Y values are the mean of triplicate reactions with standard error. The intercept lies at values for AncCR (0, −10.870).

To test the hypothesis that the evolutionary reduction in GR sensitivity was due to mutations that destabilized the receptor-hormone complex, we used a computational approach to predict the effects of historical GR mutations on the stability of AncCR. Using the AncCR:DOC crystal structure as a starting-point, we used FoldX software [43] to generate single and combination AncCR mutants, optimize the predicted structures, and calculate the predicted change in free energy of folding (ΔΔG) for mutant receptors. We calculated the distribution of stability effects for 29 single-substitution mutants and found that V43A and R116H are, as predicted, the most destabilizing substitutions, reducing stability by 1.62 and 2.81 kcal/mol, respectively (Figure 6A, Table S5). The buffering mutation C71S had a very weakly destabilizing effect (0.29 kcal/mol). The combination V43A/R116H is predicted to be extremely destabilizing in AncCR (5.31 kcal/mol); addition of C71S to this background causes a slight additional decrease in stability (5.41 kcal/mol).

We found a strong overall correlation between the predicted effects of mutations on protein stability and the observed reduction in receptor sensitivity to hormone (r^2^ = 0.78; Figure 6B, Table 1, Table S5). The relationship is even tighter (r^2^ = 0.89) when C71S-containing mutants are excluded. These results corroborate the hypothesis that V43A, R116H, and Q113K caused the evolution of reduced receptor sensitivity by destabilizing the protein-hormone complex, while C71S partially buffered the effects of these mutations through mechanisms not directly related to protein stability.

## Discussion

Our analyses allow a detailed description of the genetic, structural, and biophysical mechanisms by which AncGR1 evolved its derived function – sensitivity to only high concentrations of corticosteroid hormone – after duplication of an ancestral receptor that was sensitive to very low doses of the same hormones. The shift appears to have been driven primarily by two large-effect mutations that caused partial degradation of the receptor's structure and function. The mechanism for the functional change appears to be that these mutations compromised favorable hydrophobic interactions and hydrogen bonds in the ancestral protein, destabilizing the hormone-receptor complex. Although the combined effect of the two large-effect mutations is so great that they nearly abolish hormone sensitivity in the double mutant, a third mutation – which occurred during the same historical interval strongly buffers their effect. This buffering mutation causes no apparent effect on function in isolation; we therefore conjecture that this mutation occurred as a permissive mutation before V43A and R116H were established. Additional substitutions subsequently tuned the sensitivity of AncGR1 and its descendants. Our results do not allow us to determine the roles of selective and neutral processes in the fixation of these mutations, and the physiological significance of the GR's reduced sensitivity in the ancestral organism is unknown; it is possible, however, that the advent of a low-sensitivity GR, along with the high-sensitivity MR, allowed a greater degree of endocrine control by different doses of corticosteroids, as appears to be the case in extant elasmobranchs.

Modulating the stability of a protein:ligand complex represents one way to alter the effective dose of ligand required to produce some specific quantity of active complex. During the evolution of AncGR1, this outcome was achieved through mutations that disrupted favorable contacts among structural elements of the peptide itself, without directly affecting receptor-ligand contacts. Our findings add to a growing literature on the evolution of protein stability. Random mutations are more likely to reduce than increase protein stability [44]–[46]. Periods of mutation accumulation without purifying selection can quickly degrade proteins structures so they fall below the minimum threshold for folding and activity [13], [14], [47], [48]. Although most proteins are marginally stable, a protein's precise distance from this threshold is dynamic, depending on the specific balance of stabilizing and destabilizing mutations that have occurred [13], [49]. A stability threshold can also be relaxed by mechanisms such as the overexpression of chaperone proteins [50] or decreased selection for optimal protein function [14]. “Global suppressor” mutations that increase protein stability allow a greater number of destabilizing mutations to accumulate than would otherwise be allowed [51]–[53], including those that generate novel functions [15], [17], [49], [51], [54]–[56].

Our observations indicate that destabilizing mutations can be buffered not only by permissive mutations that increase stability but also by those that affect protein structure and function via other biophysical mechanisms, such as folding or solubility [57], [58]. The extreme reduction in stability conferred on AncGR1.1 by mutations at sites 43 and 116 are strongly buffered by historical mutation C71S, which does not affect function in isolation and is not predicted to alter protein stability. Previous studies in extant proteins have shown that introduction of a serine at the homologous site in rat and human GRs increases transcriptional activity and ligand affinity [59] and dramatically improves protein solubility and expression in bacterial cells [15], [16], [37], [40]–[42]. It has been proposed that the serine maintains the receptor in an “agonist-like” conformation, preventing the collapse and aggregation of the LBD [60]. Effects on protein folding and aggregation may explain why C71S by itself is neutral in AncCR and yet, in the context of strongly destabilizing mutations, is required to maintain the active conformation and function. An alternative explanation is that C71S may play a local role in maintaining secondary structural elements and the spatial relations between them in the active conformation; the location of C71S on H5, directly opposite where V43A packs against H5, lends credence to this possibility. Additional experiments will be necessary to directly measure the effects of mutations at sites 43, 71, and 116 on the protein's biophysical properties.

Our study highlights a creative role for partial loss-of-function mutations in the evolution of novel genes and gene functions. This aspect of GR evolution is related to the process described by Bridgham *et al*. [61] during post-duplication evolution of a different steroid receptor: in that case, a loss-of-function mutation abolished the modular LBD's ligand-activated transcriptional function and generated a competitive repressor that retained its ability to compete with its paralog for DNA and dimerization partners. The mechanism we observed during AncGR1 evolution, in contrast, involved a partial loss of activity, leading to densensitization of the receptor and a novel response to existing hormone levels. Steroid signaling relies on very precise molecular cues, and changes in receptor sensitivity can have noticeable effects on biological response [62]. After duplication of AncCR, MRs retained the ancestral receptor's sensitivity, while the evolution of reduced sensitivity in the GR created a distinctly different transcriptional regulator that responded only to high doses of hormone. These observations demonstrate how mutations that abolish or impair native protein functions can drive the evolution of novel functional roles after gene duplication [3].

## Methods

### Receptor Isolation

The little skate (*Leucoraja erinacea*) GR ligand-binding domain (LBD) was isolated previously using degenerate PCR and RACE with liver cDNA [19]. The skate GR protein sequence was used in a tblastn search of the elephant shark genome (http://esharkgenome.imcb.a-star.edu.sg/) to identify its GR LBD, and gene-specific primers were designed to amplify the coding sequence from cDNA. All other cartilaginous fish GR LBDs were isolated by hemi-degenerate PCR from cDNA using a degenerate primer in the GR DNA-binding domain (DBD) in combination with a gene-specific primer for a ∼25 bp sequence conserved in the 3′-UTR of the elephant shark and skate (5′-TCATATGCACTACATATGGTTTACAGA-3′). In total, GR LBDs were amplified using high-fidelity PCR from five cartilaginous fish species: elephant shark (*Callorhincus milii*), Atlantic sharpnose shark (*Rhizoprionodon terraenovae*), brownbanded bambooshark (*Chiloscyllium punctatum*), small-spotted catshark (*Scyliorhinus canicula*), and Atlantic stingray (*Dasyatis sabina*). Template cDNA for PCR was graciously provided by B. Venkatesh (*C. milii* and *C. punctatum*) and B.S. Nunez (*R. terraenovae*, *S. canicula*, and *D. sabina*).

### Phylogenetic Analysis

The conserved DNA- and/or ligand-binding domains of 97 steroid receptor protein sequences were aligned using Clustal X [63]. Maximum likelihood phylogenetics was performed using PhyML_aLRT [64] assuming the Jones model of evolution [65] and a four-category discrete gamma distribution of among-site rate variation, with the shape parameter estimated from the data; the JTT model was previously shown to be highly supported, with 100% posterior probability, when this and other models are compared in a Bayesian analysis [19]. Support at nodes was calculated as the chi-square statistic using an approximate likelihood ratio test [64]; the chi-square statistic represents 1-p, where p is the estimated probability that the given node would occur by chance alone.

### Ancestral Sequence Reconstruction and Gene Resurrection

The maximum likelihood tree topology differed from previously published SR phylogenies with respect to the placement of jawless fish receptors [19]; to account for this uncertainty, ancestral receptor sequences were reconstructed over both the experimental and published trees weighted by their inferred posterior probability [36]. Ancestral states were inferred using PAML version 3.15 [66] and the ancestral reconstruction tool Lazarus [36], given the sequence alignment, phylogenies, and the JTT model. For any ancestor relevant to our study, no site in the inferred sequence possessed amino acid states that differed between trees. A nucleic acid sequence coding for the LBD of the last common ancestor of all GRs (AncGR1.1) was optimized for expression in mammalian cells, synthesized *de novo* (Genscript, Piscataway, NJ), and characterized as described below.

### Tests of Evolutionary Rates

Rates of protein evolution were analyzed in HyPhy [67]. A likelihood ratio test (LRT) was used to compare the relative branch lengths from the last common GR/MR ancestor (AncCR) to the last common ancestors of all GRs (AncGR1.1) or MRs (AncMR1). Under the null hypothesis of equal rates, all branch lengths were unconstrained and optimized independently; under the alternate hypotheses, the branch leading from AncCR to AncGR1 was constrained to have the same length as that leading from AncCR to MR1 (MR in the ancestor of all jawed vertebrates). The likelihood ratio of alternate and null models was determined and a p-value calculated using a chi-squared distribution with one degree of freedom.

### Receptor Characterization

LBDs were cloned as fusion proteins into a pSG5-Gal4DBD expression vector (gift of D. Furlow) and cotransfected using Lipofectamine and Plus Reagents (Invitrogen, Carlsbad, CA) with a UAS-driven luciferase reporter gene (pFRluc) into mammalian cell culture (CHO-K1), and grown in phenol red-free α-MEM plus 10% dextran-charcoal-stripped fetal bovine serum (Hyclone, Logan, UT). Cells were incubated with transfection reagents for four hours, after which they were treated with fresh medium; after recovery, cells were treated in triplicate with hormone or vehicle control, and incubated for one day. Reporter expression was measured using Dual-Glo (Promega, Madison, WI) and dose-response relationships analyzed using Prism4 (GraphPad, La Jolla, CA). Site-directed mutagenesis was carried out using QuickChange II (Stratagene, La Jolla, CA) and clones verified by DNA sequencing. Plausible alternate states were defined as non-maximum likelihood amino acid states with posterior probability >0.20; we reasoned that residues that are present in one or more extant high-sensitivity receptors are the most likely to increase receptor sensitivity, whereas those that are present only in low-sensitivity receptors are unlikely to confer high sensitivity. Each such alternate state was introduced singly into the ML AncGR1.1, and the experimental characterization was repeated.

### Protein Growth, Purification, and X-Ray Crystallography

AncGR1.1 was subcloned into the pMCSG7-MBP-His expression vector, transformed into BL21 (DE3) pLysS cells, and grown to an OD600 of 0.8–1.0. Cultures were induced with 0.1 µM IPTG plus 50 µM of the steroid 11-deoxycorticosterone (DOC) and grown overnight at 16°C. Purification of AncGR1.1 was performed using nickel affinity chromatography and a sizing column. Pure AncGR1.1 was concentrated to 3.7 mg/mL and dialyzed into a crystallization buffer consisting of 20 mM Tris, pH 6.5, 150 mM NaCl, 5% glycerol, 50 µM CHAPS, and 50 µM hormone (DOC).

Multiple sparse matrix screens were set with AncGR1.1 protein using a Phoenix crystallization robot (Art Robbins Instruments, Sunnyvale, CA); hits formed at 22°C from the Salt Rx screen (Hampton Research, Aliso Viejo, CA). Crystals were optimized at 22°C in hanging drop diffusion plates with: 2.5–2.8 M sodium acetate trihydrate, pH 7.0, 0.1 M BIS-TRIS propane, pH 7.0, and a small peptide designed from the TIF2 Box3 steroid receptor coactivator protein. Crystals were soaked in a cryoprotectant solution containing 20% glycerol and flash-frozen in liquid nitrogen. Data was collected to 1.95 Å resolution at the South East Regional Collaborative Access Team (SER-CAT) at the Advanced Photon Source (Argonne National Laboratory) and data was processed and scaled with HKL2000 [68]. Initial phasing of the AncGR1.1 plus DOC structure was determined using molecular replacement of the AncCR with DOC (2Q3Y); model building and refinement of the structure was carried out using COOT version 0.5 [69] and REFMAC [70] in the CCP4 suite [71]. The root mean square deviation (RMSD), a measure of the overall similarity between protein backbones, was calculated using CaspR [72]. Interpretation was focused on Chain B, which displayed lower overall b-factors and had fewer crystal-packing contacts. Coordinates have been deposited in PDB with accession 3RY9.

### Analyses of Protein Stability

We used FoldX version 3.0 Beta 4 [43] to predict protein stability of AncCR and its mutational variants, using the empirical AncCR structure (PDB 2Q3Y, which contains an engineered C71S mutation to facilitate expression and crystallization) as a template. The receptor was optimized using the ‘Repair PDB’ function with sites Q39 and S76 fixed, as these side chains moved considerably in the absence of ligand (which is not modeled in FoldX). The ancestral state Cys71 was re-introduced into the AncCR sequence, and the structure was energy-minimized. GR substitutions were analyzed singly or in combination using the ‘Build Model’ function, and the change in protein stability estimated as the average difference in the free energies between the maximum likelihood and mutant AncCR structures (ΔΔG) for five runs. Several substitutions were excluded from the dataset because they generated errors (“segmentation fault” at sites Y27R, Q213K, and K246Q), bordered gaps in the electron density of the AncCR structure (A171V, K173R, and N175G), or contacted ligand (A36G).

## Supporting Information

Figure S1 Complete maximum likelihood phylogeny of steroid receptors with chi-square support statistics (blue). Support values were calculated using an approximate likelihood ratio test. The chi-square statistic represents 1-p, where p is the estimated probability that a node would occur by chance alone.
(DOC)

Figure S2 Statistical support of sites reconstructed in AncGR1.0 versus AncGR1.1. With greater taxon sampling, AncGR1.1 has a greater frequency of sites reconstructed with high statistical support (solid black, versus solid gray), and fewer sites that are ambiguously reconstructed, defined as sites with an alternate site possessing >0.20 posterior probability (open-black, versus open-gray).
(DOC)

Table S1 Log EC50 values plus standard error calculated for extant and ancestral receptors.
(DOC)

Table S2 List of sequences, species, and accession numbers used in phylogenetic analyses and ancestral reconstructions.
(DOC)

Table S3 Reconstructed sequences for AncGR1 and AncGR1.1, showing discrepancies between reconstructions, average posterior probabilities across all sites, and plausible alternate states (PP >0.20).
(DOC)

Table S4 Data collection and refinement statistics for X-ray crystallography.
(DOC)

Table S5 FoldX predictions of the change in free energy (ΔΔG) for single and combination mutants of AncCR. Values represent the average of five runs with standard deviation.
(DOC)
