# Supplementary figures and images for: Mechanisms for the Evolution of a Derived Function in the Ancestral Glucocorticoid Receptor
Source: PLoS Genet. 2011 Jun 16;7(6):e1002117. doi: 10.1371/journal.pgen.1002117 (PMC3116920; doi:10.1371/journal.pgen.1002117)

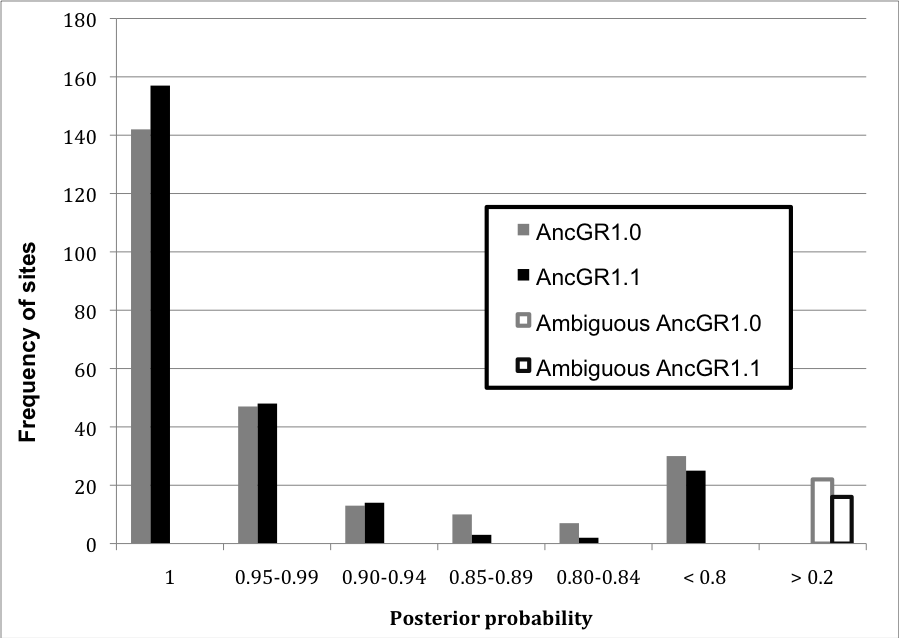

Supplement: Figure S2 — Statistical support of sites reconstructed in AncGR1.0 versus AncGR1.1. With greater taxon sampling, AncGR1.1 has a greater frequency of sites reconstructed with high statistical support (solid black, versus solid gray), and fewer sites that are ambiguously reconstructed, defined as sites with an alternate site possessing >0.20 posterior probability (open-black, versus open-gray). (DOC) [file pgen.1002117.s002.doc]
