# Supplementary material for: Mechanisms for the Evolution of a Derived Function in the Ancestral Glucocorticoid Receptor
Source: PLoS Genet. 2011 Jun 16;7(6):e1002117. doi: 10.1371/journal.pgen.1002117 (PMC3116920; doi:10.1371/journal.pgen.1002117)
Supplement: Table S1 — Log EC50 values plus standard error calculated for extant and ancestral receptors. (DOC) [file pgen.1002117.s003.doc]

|  | DOC^a^ | | B^a^ | | 1α-B^a^ | | 11-DHC^a^ | |
| --- | --- | --- | --- | --- | --- | --- | --- | --- |
| Receptor | log EC50 (M)^b^ | SE | log EC50 (M) | SE | log EC50 (M) | SE | log EC50 (M) | SE |
| Chondrichthyan GRs^c^ |  |  |  |  |  |  |  |  |
| Cmil GR | -7.761 | 0.25 | -8.196 | 0.28 | -6.438 | 0.16 | NA | NA |
| Rter GR | -7.623 | 0.12 | -7.912 | 0.16 | -6.433 | 0.12 | NA | NA |
| Cpun GR | -6.143 | 0.16 | -7.320 | 0.21 | -6.552 | 0.18 | NA | NA |
| Scan GR | -6.702 | 0.24 | -7.322 | 0.17 | -5.767 | 0.16 | NA | NA |
| Leri GR | -6.515 | 0.21 | -7.548 | 0.28 | -5.700 | 0.13 | NA | NA |
| Dsab GR | NA^d^ | NA | NA | NA | NA | NA | NA | NA |
|  |  |  |  |  |  |  |  |  |
| Ancestral reconstructions |  |  |  |  |  |  |  |  |
| AncCR | -10.870 | 0.34 | -10.410 | 0.29 | -7.992 | 0.16 | -7.315 | 0.17 |
| AncGR1.0 | -9.182 | 0.20 | -8.944 | 0.15 | -7.735 | 0.19 | -7.304 | 0.12 |
| AncGR1.1 | -8.144 | 0.20 | -8.151 | 0.16 | -6.232 | 0.09 | -5.924 | 0.14 |
|  |  |  |  |  |  |  |  |  |
| Alternate reconstructions of AncGR1.1^d^ |  |  |  |  |  |  |  |  |
| T25S | -8.245 | 0.32 |  |  |  |  |  |  |
| E110D | -8.196 | 0.25 |  |  |  |  |  |  |
| K113Q | -7.993 | 0.21 |  |  |  |  |  |  |
| H116Q | -8.972 | 0.22 |  |  |  |  |  |  |
| T214S | -8.159 | 0.18 |  |  |  |  |  |  |
| T214A | -8.137 | 0.28 |  |  |  |  |  |  |

^a^ Hormones: DOC, 11-deoxycorticosterone; B, corticosterone; 1α-B, 1α-hydroxycorticosterone; 11-DHC, 11-dehydrocorticosterone

^b^ The concentration of hormone for half-maximal activation, calculated by triplicate reactions in a reporter gene assay

^c^ Cartilaginous fish species listed in Fig. 1

^d^ NA defined as < 2-fold activation for EC_50_ > 1μM of hormone

^e^ Plausible alternate states have a posterior probability > 0.20 and are present in other high-sensitivity corticosteroid receptors
