# Supplementary material for: Mechanisms for the Evolution of a Derived Function in the Ancestral Glucocorticoid Receptor
Source: PLoS Genet. 2011 Jun 16;7(6):e1002117. doi: 10.1371/journal.pgen.1002117 (PMC3116920; doi:10.1371/journal.pgen.1002117)
Supplement: Table S2 — List of sequences, species, and accession numbers used in phylogenetic analyses and ancestral reconstructions. (DOC) [file pgen.1002117.s004.doc]

| Sequence | Species | Common name | Accession No. |
| --- | --- | --- | --- |
| TakrubAR | Tetraodon nigroviridis | green-spotted pufferfish | CAG08385.1 |
| GamAffARa | Gambusia affinis | western mosquitofish | BAD81045.1 |
| HapBurARb | Haplochromis burtoni | African cichlid | AAD25074.2 |
| OreNilARa | Oreochromis niloticus | Nile tilapia | BAB20081.1 |
| LeuEriAR | Leucoraja erinacea | little skate | ABD46746.1 |
| SquAcaAr | Squalus acanthias | spiny dogfish | AAP55843.2 |
| SusScrAR | Sus scrofa | pig | Q9GKL7.2 |
| CrocroAR | Crocuta crocuta | Spotted hyena | Q8MIK0.1 |
| HomSapAR | Homo sapiens | human | NP_000035.2 |
| CanFamAR | Canis lupus familiaris | dog | NP_001003053.1 |
| OryCunAR | Oryzias latipes | Japanese ricefish | ABV55993.1 |
| RatNovAR | Rattus norvegicus | rat | NP_036634.1 |
| CotJapAR | Coturnix japonica | Japanese quail | BAD38679.1 |
| SerCanAR | Serinus canaria | canary | AAA17402.1 |
| TaeGutAR | Taeniopygia guttata | zebrafinch | NP_001070156.1 |
| ManVitAR | Manacus vitellinus | golden-collared manakin | AAY42038.1 |
| RanCatAR | Rana catesbeiana | bullfrog | Q7T1K4.1 |
| XenLaeAR | Xenopus laevis | African clawed toad | AAI70347.1 |
| EngPusAR | Engystomops pustulosus | túngara frog | ABC49718.1 |
| AngJapARa | Anguilla japonica | Japanese eel | BAA75464.1 |
| AngJapARb | Anguilla japonica | Japanese eel | BAA83805.1 |
| HapBurARb | Haplochromis burtoni | African cichlid | AAL92878.2 |
| OreNilARb | Oreochromis niloticus | Nile tilapia | BAB20082.1 |
| OryLatARb | Oryzias latipes | Japanese ricefish | NP_001098151.1 |
| GamAffARa | Gambusia affinis | western mosquitofish | BAD52084.1 |
| KryMarAR | Kryptolebias marmoratus | mangrove rivulus | ABC68612.1 |
| HalTriAR | Halichoeres trimaculatus | protogynous wrasse | AAG48340.1 |
| GasAcuARb2 | Gasterosteus aculeatus | three-spined stickleback | AAO83572.1 |
| AcaSchAR | Acanthopagrus schlegelii | black porgy | AAO61694.1 |
| MicUndAR | Micropogonias undulatus | Atlantic croaker | AAU09477.1 |
| PagMajAR | Pagrus major | red sea bream | BAA33451.1 |
| DicLabAR | Dicentrarchus labrax | European sea bass | AAT76433.1 |
| DanRerAR | Danio rerio | zebrafish | ABO47800.1 |
| CarAurAR | Carassius auratus | goldfish | AAM09278.1 |
| PimProAR | Pimephales promelas | fathead minnow | AAF88138.2 |
| OncMykARa | Oncorhynchus mykiss | rainbow trout | NP_001117656.1 |
| OncMykARb | Oncorhynchus mykiss | rainbow trout | NP_001117657.1 |
| OncMykMR | Oncorhynchus mykiss | rainbow trout | NP_001117955.1 |
| HapBurMR | Haplochromis burtoni | African cichlid | AAM27890.1 |
| XenLaeMR | Xenopus laevis | African clawed toad | NP_001084074.1 |
| OviAriMR | Ovis aries | sheep | Q9BDJ7.1 |
| GalGalMR | Gallus gallus | chicken | ACO37437.1 |
| TaeGutMR | Taeniopygia guttata | zebrafinch | NP_001070158.1 |
| TupBelMR | Tupaia belangeri | northern tree shrew | Q29131.1 |
| RatRatMR | Rattus norvegicus | rat | NP_037263.1 |
| MusMusMR | Mus musculus | mouse | Q8VII8.2 |
| SaiBolMR | Saimiri boliviensis | squirrel monkey | Q4JM28.1 |
| CanFamMR | Canis lupus familiaris | dog | XP_532685.2 |
| HomSapMR | Homo sapiens | human | P08235.1 |
| SusScrMR | Sus scrofa | pig | AAB53273 |
| CalMilMR | Callorhinchus milii | elephant shark | PREDICTED^*^ |
| LeuEriMR | Leucoraja erinacea | little skate | ABD46745.1 |
| OncMykGR | Oncorhynchus mykiss | rainbow trout | NP_001118202.1 |
| SalTruGR | Salmo trutta | brown trout | AAW56453.1 |
| HapBurGR2a | Haplochromis burtoni | African cichlid | AAM27888.1 |
| ParOliGR | Paralichthys olivaceus | Japanese flounder | O73673.1 |
| OncMykGR2 | Oncorhynchus mykiss | rainbow trout | NP_001117954.1 |
| DanRerGR | Danio rerio | zebrafish | NP_001018547.2 |
| PimProGR | Pimephales promelas | fathead minnow | AAT02177.1 |
| HapBurGR | Haplochromis burtoni | African cichlid | AAM27887.1 |
| DicLabGR | Dicentrarchus labrax | European sea bass | AAT41627.1 |
| SpaAurGR | Sparus aurata | gilthead sea bream | ABF30967.1 |
| XenLaeGR | Xenopus laevis | African clawed toad | P49844.1 |
| CavPorGR | Cavia porcellus | guinea pig | P49115.1 |
| AllMissGR | Alligator mississippiensis | alligator | AAM93197.1 |
| GalGalGR | Gallus gallus | chicken | NP_001032915.1 |
| TupBelGR | Tupaia belangeri | northern tree shrew | Q95267.1 |
| SusSusGR | Sus scrofa | pig | NP_001008481.1 |
| PanTroGR | Pan troglodytes | chimp | XP_527059.1 |
| SaiBolGR | Saimiri boliviensis boliviensis | Bolivian squirrel monkey | O13186.1 |
| BosTauGR | Bos taurus | cow | AAO85271.2 |
| OrtCunGR | Oryctolagus cuniculus | European rabbit | NP_001075616.1 |
| CanFamGR | Canis lupus familiaris | dog | XP_535225.2 |
| MusMusGR | Mus musculus | mouse | P06537.1 |
| RatRatGR | Rattus norvegicus | rat | P06536.2 |
| CalMilSGR | Callorhinchus milii | elephant shark | AEF12274 |
| DasSabGR | Dasyatis sabina | Atlantic stingray | AEF12278 |
| LeuEriGR | Leucoraja erinacea | Little skate | ABD46744.1 |
| RhiTerGR | Rhizoprionodon terraenovae | Atlantic sharpnose shark | AEF12275 |
| ChiPunGR | Chiloscyllium punctatum | brownbanded bambooshark | AEF12276 |
| ScyCanGR | Scyliorhinus canicula | small-spotted catshark | AEF12277 |
| PetMarCR | Petromyzon marinus | sea lamprey | AAK20930.1 |
| MyxGluCR | Myxine glutinosa | hagfish | ABD46742.1 |
| PetMarPR | Petromyzon marinus | sea lamprey | AAK20931.1 |
| MyxGluSR | Myxine glutinosa | hagfish | ABD46743.1 |
| CalMilPR | Callorhinchus milii | elephant shark | PREDICTED^*^ |
| LeuEriPR | Leucoraja erinacea | little skate | ABD46747.1 |
| RanDybPR | Rana dybowskii | Korean brown frog | Q8AYI2.1 |
| XenLaePR | Xenopus laevis | African clawed toad | NP_001079100.1 |
| CanFamPR | Canis lupus familiaris | dog | AAG09282.1 |
| OrtCunPR | Oryctolagus cuniculus | European rabbit | NP_001075736.1 |
| HomSapPR | Homo sapiens | human | AAD01587.1 |
| MusMusPR | Mus musculus | mouse | Q00175.1 |
| RatRatPR | Rattus norvegicus | rat | NP_074038.1 |
| AspInoPR | Aspidoscelis inornata | little striped whiptail | ACJ45777.2 |
| AllMisPR | Alligator mississippiensis | alligator | BAD08350.1 |
| GalGalPR | Gallus gallus | chicken | NP_990593.1 |

^*^ Predicted sequence from the elephant shark genome project (<http://esharkgenome.imcb.a-star.edu.sg/>)
