# Supplementary material for: Mechanisms for the Evolution of a Derived Function in the Ancestral Glucocorticoid Receptor
Source: PLoS Genet. 2011 Jun 16;7(6):e1002117. doi: 10.1371/journal.pgen.1002117 (PMC3116920; doi:10.1371/journal.pgen.1002117)
Supplement: Table S3 — Reconstructed sequences for AncGR1 and AncGR1.1, showing discrepancies between reconstructions, average posterior probabilities across all sites, and plausible alternate states (PP >0.20). (DOC) [file pgen.1002117.s005.doc]

| AncGR1 Sequence  (Accession: ABU96169) | | | |  |  |  |  | AncGR1.1 Sequence  (Acession: AEF12279) | | | |  |  |  |
| --- | --- | --- | --- | --- | --- | --- | --- | --- | --- | --- | --- | --- | --- | --- |
| Site # | ML St. | PP | 2^nd^ best | PP of alt state | 3^rd^ best | PP of alt state | Diff | Site # | ML St. | PP | 2^nd^ best | PP of alt state | 3^rd^ best | PP of alt state |
| -2 | P | 1.00 |  |  |  |  |  | -2 | P | 1.00 |  |  |  |  |
| -1 | S | 0.81 |  |  |  |  | X | -1 | T | 0.39 | S | 0.34 | A | 0.26 |
| 1 | L | 0.74 |  |  |  |  | X | 1 | M | 0.86 |  |  |  |  |
| 2 | I | 0.62 | V | 0.28 |  |  |  | 2 | I | 0.93 |  |  |  |  |
| 3 | S | 1.00 |  |  |  |  |  | 3 | S | 1.00 |  |  |  |  |
| 4 | I | 0.87 |  |  |  |  |  | 4 | I | 0.95 |  |  |  |  |
| 5 | L | 1.00 |  |  |  |  |  | 5 | L | 1.00 |  |  |  |  |
| 6 | E | 1.00 |  |  |  |  |  | 6 | E | 1.00 |  |  |  |  |
| 7 | V | 0.37 |  |  |  |  | X | 7 | A | 0.72 | V | 0.24 |  |  |
| 8 | I | 1.00 |  |  |  |  |  | 8 | I | 1.00 |  |  |  |  |
| 9 | E | 1.00 |  |  |  |  |  | 9 | E | 1.00 |  |  |  |  |
| 10 | P | 1.00 |  |  |  |  |  | 10 | P | 1.00 |  |  |  |  |
| 11 | E | 0.89 |  |  |  |  | X | 11 | D | 0.79 | E | 0.21 |  |  |
| 12 | V | 0.90 |  |  |  |  |  | 12 | V | 0.99 |  |  |  |  |
| 13 | L | 0.65 | V | 0.22 |  |  | X | 13 | I | 0.83 |  |  |  |  |
| 14 | Y | 1.00 |  |  |  |  |  | 14 | Y | 1.00 |  |  |  |  |
| 15 | A | 0.96 |  |  |  |  |  | 15 | A | 1.00 |  |  |  |  |
| 16 | G | 1.00 |  |  |  |  |  | 16 | G | 1.00 |  |  |  |  |
| 17 | Y | 1.00 |  |  |  |  |  | 17 | Y | 1.00 |  |  |  |  |
| 18 | D | 1.00 |  |  |  |  |  | 18 | D | 1.00 |  |  |  |  |
| 19 | S | 0.88 |  |  |  |  |  | 19 | S | 0.97 |  |  |  |  |
| 20 | S | 0.75 | T | 0.23 |  |  | X | 20 | T | 0.97 |  |  |  |  |
| 21 | L | 0.97 |  |  |  |  |  | 21 | L | 0.92 |  |  |  |  |
| 22 | P | 1.00 |  |  |  |  |  | 22 | P | 1.00 |  |  |  |  |
| 23 | D | 1.00 |  |  |  |  |  | 23 | D | 1.00 |  |  |  |  |
| 24 | T | 1.00 |  |  |  |  |  | 24 | T | 1.00 |  |  |  |  |
| 25 | T | 0.54 | S | 0.32 |  |  |  | 25 | T | 0.52 | S | 0.27 |  |  |
| 26 | N | 0.93 |  |  |  |  |  | 26 | N | 0.91 |  |  |  |  |
| 27 | R | 0.88 |  |  |  |  |  | 27 | R | 0.96 |  |  |  |  |
| 28 | L | 1.00 |  |  |  |  |  | 28 | L | 1.00 |  |  |  |  |
| 29 | L | 0.97 |  |  |  |  |  | 29 | L | 0.99 |  |  |  |  |
| 30 | S | 0.98 |  |  |  |  |  | 30 | S | 0.99 |  |  |  |  |
| 31 | S | 0.85 |  |  |  |  |  | 31 | S | 0.77 |  |  |  |  |
| 32 | L | 1.00 |  |  |  |  |  | 32 | L | 1.00 |  |  |  |  |
| 33 | N | 1.00 |  |  |  |  |  | 33 | N | 1.00 |  |  |  |  |
| 34 | R | 0.91 |  |  |  |  |  | 34 | R | 0.90 |  |  |  |  |
| 35 | L | 1.00 |  |  |  |  |  | 35 | L | 1.00 |  |  |  |  |
| 36 | G | 0.98 |  |  |  |  |  | 36 | G | 1.00 |  |  |  |  |
| 37 | G | 0.99 |  |  |  |  |  | 37 | G | 1.00 |  |  |  |  |
| 38 | R | 0.72 |  |  |  |  |  | 38 | R | 0.97 |  |  |  |  |
| 39 | Q | 1.00 |  |  |  |  |  | 39 | Q | 1.00 |  |  |  |  |
| 40 | M | 0.99 |  |  |  |  |  | 40 | M | 0.99 |  |  |  |  |
| 41 | V | 0.82 |  |  |  |  | X | 41 | I | 0.70 | V | 0.30 |  |  |
| 42 | S | 0.90 |  |  |  |  |  | 42 | S | 0.99 |  |  |  |  |
| 43 | V | 0.98 |  |  |  |  | X | 43 | A | 0.55 | V | 0.45 |  |  |
| 44 | V | 1.00 |  |  |  |  |  | 44 | V | 1.00 |  |  |  |  |
| 45 | K | 1.00 |  |  |  |  |  | 45 | K | 1.00 |  |  |  |  |
| 46 | W | 1.00 |  |  |  |  |  | 46 | W | 1.00 |  |  |  |  |
| 47 | A | 1.00 |  |  |  |  |  | 47 | A | 1.00 |  |  |  |  |
| 48 | K | 1.00 |  |  |  |  |  | 48 | K | 1.00 |  |  |  |  |
| 49 | A | 0.84 |  |  |  |  |  | 49 | A | 0.78 |  |  |  |  |
| 50 | L | 1.00 |  |  |  |  |  | 50 | L | 0.94 |  |  |  |  |
| 51 | P | 1.00 |  |  |  |  |  | 51 | P | 1.00 |  |  |  |  |
| 52 | G | 1.00 |  |  |  |  |  | 52 | G | 1.00 |  |  |  |  |
| 53 | F | 1.00 |  |  |  |  |  | 53 | F | 1.00 |  |  |  |  |
| 54 | R | 1.00 |  |  |  |  |  | 54 | R | 1.00 |  |  |  |  |
| 55 | N | 0.96 |  |  |  |  |  | 55 | N | 1.00 |  |  |  |  |
| 56 | L | 1.00 |  |  |  |  |  | 56 | L | 1.00 |  |  |  |  |
| 57 | H | 1.00 |  |  |  |  |  | 57 | H | 1.00 |  |  |  |  |
| 58 | L | 1.00 |  |  |  |  |  | 58 | L | 1.00 |  |  |  |  |
| 59 | D | 1.00 |  |  |  |  |  | 59 | D | 1.00 |  |  |  |  |
| 60 | D | 1.00 |  |  |  |  |  | 60 | D | 1.00 |  |  |  |  |
| 61 | Q | 1.00 |  |  |  |  |  | 61 | Q | 1.00 |  |  |  |  |
| 62 | M | 1.00 |  |  |  |  |  | 62 | M | 1.00 |  |  |  |  |
| 63 | T | 0.99 |  |  |  |  |  | 63 | T | 0.98 |  |  |  |  |
| 64 | L | 1.00 |  |  |  |  |  | 64 | L | 1.00 |  |  |  |  |
| 65 | L | 0.98 |  |  |  |  |  | 65 | L | 0.99 |  |  |  |  |
| 66 | Q | 1.00 |  |  |  |  |  | 66 | Q | 1.00 |  |  |  |  |
| 67 | Y | 1.00 |  |  |  |  |  | 67 | Y | 1.00 |  |  |  |  |
| 68 | S | 1.00 |  |  |  |  |  | 68 | S | 1.00 |  |  |  |  |
| 69 | W | 1.00 |  |  |  |  |  | 69 | W | 1.00 |  |  |  |  |
| 70 | M | 1.00 |  |  |  |  |  | 70 | M | 1.00 |  |  |  |  |
| 71 | S | 0.46 | C | 0.38 |  |  |  | 71 | S | 0.76 |  |  |  |  |
| 72 | L | 1.00 |  |  |  |  |  | 72 | L | 1.00 |  |  |  |  |
| 73 | M | 1.00 |  |  |  |  |  | 73 | M | 1.00 |  |  |  |  |
| 74 | A | 0.72 |  |  |  |  |  | 74 | A | 0.59 |  |  |  |  |
| 75 | F | 1.00 |  |  |  |  |  | 75 | F | 1.00 |  |  |  |  |
| 76 | S | 0.81 |  |  |  |  |  | 76 | S | 0.84 |  |  |  |  |
| 77 | L | 1.00 |  |  |  |  |  | 77 | L | 1.00 |  |  |  |  |
| 78 | G | 0.86 |  |  |  |  |  | 78 | G | 1.00 |  |  |  |  |
| 79 | W | 1.00 |  |  |  |  |  | 79 | W | 1.00 |  |  |  |  |
| 80 | R | 1.00 |  |  |  |  |  | 80 | R | 1.00 |  |  |  |  |
| 81 | S | 1.00 |  |  |  |  |  | 81 | S | 1.00 |  |  |  |  |
| 82 | Y | 1.00 |  |  |  |  |  | 82 | Y | 1.00 |  |  |  |  |
| 83 | K | 0.98 |  |  |  |  | X | 83 | Q | 0.68 | K | 0.31 |  |  |
| 84 | H | 0.98 |  |  |  |  |  | 84 | H | 0.99 |  |  |  |  |
| 85 | S | 0.66 | T | 0.32 |  |  | X | 85 | T | 0.98 |  |  |  |  |
| 86 | N | 1.00 |  |  |  |  |  | 86 | N | 1.00 |  |  |  |  |
| 87 | G | 0.97 |  |  |  |  |  | 87 | G | 0.95 |  |  |  |  |
| 88 | N | 0.50 | S | 0.28 |  |  |  | 88 | N | 0.97 |  |  |  |  |
| 89 | M | 1.00 |  |  |  |  |  | 89 | M | 1.00 |  |  |  |  |
| 90 | L | 1.00 |  |  |  |  |  | 90 | L | 1.00 |  |  |  |  |
| 91 | Y | 0.79 |  |  |  |  |  | 91 | Y | 0.62 | F | 0.32 |  |  |
| 92 | F | 1.00 |  |  |  |  |  | 92 | F | 1.00 |  |  |  |  |
| 93 | A | 1.00 |  |  |  |  |  | 93 | A | 1.00 |  |  |  |  |
| 94 | P | 1.00 |  |  |  |  |  | 94 | P | 1.00 |  |  |  |  |
| 95 | D | 1.00 |  |  |  |  |  | 95 | D | 1.00 |  |  |  |  |
| 96 | L | 1.00 |  |  |  |  |  | 96 | L | 1.00 |  |  |  |  |
| 97 | I | 0.89 |  |  |  |  |  | 97 | I | 0.97 |  |  |  |  |
| 98 | F | 1.00 |  |  |  |  |  | 98 | F | 0.97 |  |  |  |  |
| 99 | N | 1.00 |  |  |  |  |  | 99 | N | 1.00 |  |  |  |  |
| 100 | E | 1.00 |  |  |  |  |  | 100 | E | 1.00 |  |  |  |  |
| 101 | E | 0.89 |  |  |  |  |  | 101 | E | 0.91 |  |  |  |  |
| 102 | R | 1.00 |  |  |  |  |  | 102 | R | 1.00 |  |  |  |  |
| 103 | M | 1.00 |  |  |  |  |  | 103 | M | 1.00 |  |  |  |  |
| 104 | Q | 0.97 |  |  |  |  |  | 104 | Q | 0.97 |  |  |  |  |
| 105 | Q | 0.97 |  |  |  |  |  | 105 | Q | 0.98 |  |  |  |  |
| 106 | S | 0.99 |  |  |  |  |  | 106 | S | 1.00 |  |  |  |  |
| 107 | A | 0.48 | T | 0.36 |  |  | X | 107 | S | 0.79 |  |  |  |  |
| 108 | M | 1.00 |  |  |  |  |  | 108 | M | 1.00 |  |  |  |  |
| 109 | Y | 1.00 |  |  |  |  |  | 109 | Y | 1.00 |  |  |  |  |
| 110 | D | 0.88 |  |  |  |  | X | 110 | E | 0.70 | D | 0.30 |  |  |
| 111 | L | 0.99 |  |  |  |  |  | 111 | L | 1.00 |  |  |  |  |
| 112 | C | 1.00 |  |  |  |  |  | 112 | C | 1.00 |  |  |  |  |
| 113 | Q | 0.58 | K | 0.33 |  |  | X | 113 | K | 0.65 | Q | 0.28 |  |  |
| 114 | G | 0.97 |  |  |  |  |  | 114 | G | 0.99 |  |  |  |  |
| 115 | M | 1.00 |  |  |  |  |  | 115 | M | 1.00 |  |  |  |  |
| 116 | R | 0.41 | Q | 0.27 |  |  | X | 116 | H | 0.47 | Q | 0.28 |  |  |
| 117 | K | 0.49 | Q | 0.32 |  |  |  | 117 | K | 0.46 | Q | 0.22 | N | 0.20 |
| 118 | I | 0.99 |  |  |  |  |  | 118 | I | 1.00 |  |  |  |  |
| 119 | S | 0.96 |  |  |  |  |  | 119 | S | 0.97 |  |  |  |  |
| 120 | V | 0.28 | S | 0.21 |  |  | X | 120 | L | 0.42 | I | 0.23 |  |  |
| 121 | E | 0.95 |  |  |  |  |  | 121 | E | 1.00 |  |  |  |  |
| 122 | F | 1.00 |  |  |  |  |  | 122 | F | 1.00 |  |  |  |  |
| 123 | V | 0.74 |  |  |  |  |  | 123 | V | 0.74 |  |  |  |  |
| 124 | R | 0.92 |  |  |  |  |  | 124 | R | 0.89 |  |  |  |  |
| 125 | L | 1.00 |  |  |  |  |  | 125 | L | 1.00 |  |  |  |  |
| 126 | Q | 0.99 |  |  |  |  |  | 126 | Q | 1.00 |  |  |  |  |
| 127 | V | 0.99 |  |  |  |  |  | 127 | V | 1.00 |  |  |  |  |
| 128 | T | 0.47 | S | 0.31 |  |  | X | 128 | S | 0.86 |  |  |  |  |
| 129 | Y | 0.95 |  |  |  |  |  | 129 | Y | 0.96 |  |  |  |  |
| 130 | E | 0.92 |  |  |  |  |  | 130 | E | 0.97 |  |  |  |  |
| 131 | E | 1.00 |  |  |  |  |  | 131 | E | 1.00 |  |  |  |  |
| 132 | Y | 0.98 |  |  |  |  |  | 132 | Y | 0.58 | F | 0.42 |  |  |
| 133 | L | 1.00 |  |  |  |  |  | 133 | L | 1.00 |  |  |  |  |
| 134 | C | 1.00 |  |  |  |  |  | 134 | C | 1.00 |  |  |  |  |
| 135 | M | 1.00 |  |  |  |  |  | 135 | M | 1.00 |  |  |  |  |
| 136 | K | 1.00 |  |  |  |  |  | 136 | K | 1.00 |  |  |  |  |
| 137 | V | 0.74 | A | 0.26 |  |  |  | 137 | V | 0.63 | A | 0.34 |  |  |
| 138 | L | 1.00 |  |  |  |  |  | 138 | L | 1.00 |  |  |  |  |
| 139 | L | 1.00 |  |  |  |  |  | 139 | L | 1.00 |  |  |  |  |
| 140 | L | 1.00 |  |  |  |  |  | 140 | L | 1.00 |  |  |  |  |
| 141 | L | 1.00 |  |  |  |  |  | 141 | L | 1.00 |  |  |  |  |
| 142 | S | 0.98 |  |  |  |  |  | 142 | S | 1.00 |  |  |  |  |
| 143 | T | 1.00 |  |  |  |  |  | 143 | T | 1.00 |  |  |  |  |
| 144 | V | 0.76 | I | 0.25 |  |  |  | 144 | V | 0.90 |  |  |  |  |
| 145 | P | 1.00 |  |  |  |  |  | 145 | P | 1.00 |  |  |  |  |
| 146 | K | 0.99 |  |  |  |  |  | 146 | K | 0.95 |  |  |  |  |
| 147 | D | 0.94 |  |  |  |  |  | 147 | D | 0.99 |  |  |  |  |
| 148 | G | 1.00 |  |  |  |  |  | 148 | G | 1.00 |  |  |  |  |
| 149 | L | 1.00 |  |  |  |  |  | 149 | L | 1.00 |  |  |  |  |
| 150 | K | 1.00 |  |  |  |  |  | 150 | K | 1.00 |  |  |  |  |
| 151 | S | 1.00 |  |  |  |  |  | 151 | S | 1.00 |  |  |  |  |
| 152 | Q | 1.00 |  |  |  |  |  | 152 | Q | 1.00 |  |  |  |  |
| 153 | A | 0.99 |  |  |  |  |  | 153 | A | 1.00 |  |  |  |  |
| 154 | T | 0.37 | A | 0.23 |  |  | X | 154 | A | 0.68 |  |  |  |  |
| 155 | F | 1.00 |  |  |  |  |  | 155 | F | 1.00 |  |  |  |  |
| 156 | D | 1.00 |  |  |  |  |  | 156 | D | 0.97 |  |  |  |  |
| 157 | E | 1.00 |  |  |  |  |  | 157 | E | 1.00 |  |  |  |  |
| 158 | I | 0.96 |  |  |  |  |  | 158 | I | 0.99 |  |  |  |  |
| 159 | R | 1.00 |  |  |  |  |  | 159 | R | 1.00 |  |  |  |  |
| 160 | M | 0.98 |  |  |  |  |  | 160 | M | 0.90 |  |  |  |  |
| 161 | S | 0.51 | N | 0.31 |  |  |  | 161 | S | 0.92 |  |  |  |  |
| 162 | Y | 1.00 |  |  |  |  |  | 162 | Y | 1.00 |  |  |  |  |
| 163 | I | 1.00 |  |  |  |  |  | 163 | I | 1.00 |  |  |  |  |
| 164 | K | 1.00 |  |  |  |  |  | 164 | K | 1.00 |  |  |  |  |
| 165 | E | 1.00 |  |  |  |  |  | 165 | E | 1.00 |  |  |  |  |
| 166 | L | 1.00 |  |  |  |  |  | 166 | L | 1.00 |  |  |  |  |
| 167 | G | 0.97 |  |  |  |  |  | 167 | G | 0.99 |  |  |  |  |
| 168 | K | 0.95 |  |  |  |  |  | 168 | K | 0.98 |  |  |  |  |
| 169 | A | 0.97 |  |  |  |  |  | 169 | A | 0.99 |  |  |  |  |
| 170 | I | 0.99 |  |  |  |  |  | 170 | I | 1.00 |  |  |  |  |
| 171 | V | 0.87 |  |  |  |  |  | 171 | V | 0.97 |  |  |  |  |
| 172 | K | 0.96 |  |  |  |  |  | 172 | K | 0.97 |  |  |  |  |
| 173 | K | 0.57 | R | 0.41 |  |  | X | 173 | R | 0.78 | K | 0.20 |  |  |
| 174 | E | 0.99 |  |  |  |  |  | 174 | E | 1.00 |  |  |  |  |
| 175 | G | 0.74 |  |  |  |  |  | 175 | G | 0.93 |  |  |  |  |
| 176 | N | 1.00 |  |  |  |  |  | 176 | N | 1.00 |  |  |  |  |
| 177 | S | 0.98 |  |  |  |  |  | 177 | S | 0.93 |  |  |  |  |
| 178 | S | 0.90 |  |  |  |  |  | 178 | S | 0.97 |  |  |  |  |
| 179 | Q | 1.00 |  |  |  |  |  | 179 | Q | 1.00 |  |  |  |  |
| 180 | N | 0.96 |  |  |  |  |  | 180 | N | 0.99 |  |  |  |  |
| 181 | W | 1.00 |  |  |  |  |  | 181 | W | 1.00 |  |  |  |  |
| 182 | Q | 0.98 |  |  |  |  |  | 182 | Q | 1.00 |  |  |  |  |
| 183 | R | 1.00 |  |  |  |  |  | 183 | R | 1.00 |  |  |  |  |
| 184 | F | 1.00 |  |  |  |  |  | 184 | F | 1.00 |  |  |  |  |
| 185 | Y | 1.00 |  |  |  |  |  | 185 | Y | 1.00 |  |  |  |  |
| 186 | Q | 1.00 |  |  |  |  |  | 186 | Q | 1.00 |  |  |  |  |
| 187 | L | 1.00 |  |  |  |  |  | 187 | L | 1.00 |  |  |  |  |
| 188 | T | 1.00 |  |  |  |  |  | 188 | T | 1.00 |  |  |  |  |
| 189 | K | 1.00 |  |  |  |  |  | 189 | K | 1.00 |  |  |  |  |
| 190 | L | 1.00 |  |  |  |  |  | 190 | L | 1.00 |  |  |  |  |
| 191 | L | 1.00 |  |  |  |  |  | 191 | L | 0.99 |  |  |  |  |
| 192 | D | 1.00 |  |  |  |  |  | 192 | D | 1.00 |  |  |  |  |
| 193 | S | 1.00 |  |  |  |  |  | 193 | S | 1.00 |  |  |  |  |
| 194 | M | 1.00 |  |  |  |  |  | 194 | M | 1.00 |  |  |  |  |
| 195 | H | 1.00 |  |  |  |  |  | 195 | H | 1.00 |  |  |  |  |
| 196 | D | 0.84 |  |  |  |  |  | 196 | D | 0.92 |  |  |  |  |
| 197 | L | 0.94 |  |  |  |  |  | 197 | L | 0.95 |  |  |  |  |
| 198 | V | 1.00 |  |  |  |  |  | 198 | V | 1.00 |  |  |  |  |
| 199 | G | 0.69 | E | 0.28 |  |  |  | 199 | G | 0.99 |  |  |  |  |
| 200 | G | 0.97 |  |  |  |  |  | 200 | G | 1.00 |  |  |  |  |
| 201 | L | 1.00 |  |  |  |  |  | 201 | L | 1.00 |  |  |  |  |
| 202 | L | 1.00 |  |  |  |  |  | 202 | L | 1.00 |  |  |  |  |
| 203 | Q | 0.96 |  |  |  |  |  | 203 | Q | 0.97 |  |  |  |  |
| 204 | F | 1.00 |  |  |  |  |  | 204 | F | 1.00 |  |  |  |  |
| 205 | C | 1.00 |  |  |  |  |  | 205 | C | 1.00 |  |  |  |  |
| 206 | F | 1.00 |  |  |  |  |  | 206 | F | 1.00 |  |  |  |  |
| 207 | Y | 1.00 |  |  |  |  |  | 207 | Y | 1.00 |  |  |  |  |
| 208 | T | 1.00 |  |  |  |  |  | 208 | T | 1.00 |  |  |  |  |
| 209 | F | 1.00 |  |  |  |  |  | 209 | F | 1.00 |  |  |  |  |
| 210 | V | 0.80 |  |  |  |  |  | 210 | V | 0.98 |  |  |  |  |
| 211 | Q | 0.82 |  |  |  |  | X | 211 | E | 0.98 |  |  |  |  |
| 212 | S | 1.00 |  |  |  |  |  | 212 | S | 1.00 |  |  |  |  |
| 213 | K | 0.95 |  |  |  |  |  | 213 | K | 0.99 |  |  |  |  |
| 214 | T | 0.42 | A | 0.36 |  |  |  | 214 | T | 0.53 | A | 0.24 | S | 0.22 |
| 215 | L | 1.00 |  |  |  |  |  | 215 | L | 1.00 |  |  |  |  |
| 216 | S | 0.99 |  |  |  |  |  | 216 | S | 1.00 |  |  |  |  |
| 217 | V | 1.00 |  |  |  |  |  | 217 | V | 1.00 |  |  |  |  |
| 218 | E | 1.00 |  |  |  |  |  | 218 | E | 1.00 |  |  |  |  |
| 219 | F | 1.00 |  |  |  |  |  | 219 | F | 1.00 |  |  |  |  |
| 220 | P | 1.00 |  |  |  |  |  | 220 | P | 1.00 |  |  |  |  |
| 221 | E | 1.00 |  |  |  |  |  | 221 | E | 1.00 |  |  |  |  |
| 222 | M | 1.00 |  |  |  |  |  | 222 | M | 1.00 |  |  |  |  |
| 223 | L | 1.00 |  |  |  |  |  | 223 | L | 1.00 |  |  |  |  |
| 224 | V | 0.68 | A | 0.31 |  |  |  | 224 | V | 0.90 |  |  |  |  |
| 225 | E | 1.00 |  |  |  |  |  | 225 | E | 1.00 |  |  |  |  |
| 226 | I | 1.00 |  |  |  |  |  | 226 | I | 1.00 |  |  |  |  |
| 227 | I | 1.00 |  |  |  |  |  | 227 | I | 1.00 |  |  |  |  |
| 228 | S | 1.00 |  |  |  |  |  | 228 | S | 1.00 |  |  |  |  |
| 229 | N | 0.96 |  |  |  |  |  | 229 | N | 0.99 |  |  |  |  |
| 230 | Q | 1.00 |  |  |  |  |  | 230 | Q | 1.00 |  |  |  |  |
| 231 | L | 1.00 |  |  |  |  |  | 231 | L | 1.00 |  |  |  |  |
| 232 | P | 1.00 |  |  |  |  |  | 232 | P | 1.00 |  |  |  |  |
| 233 | K | 1.00 |  |  |  |  |  | 233 | K | 1.00 |  |  |  |  |
| 234 | V | 0.90 |  |  |  |  |  | 234 | V | 0.98 |  |  |  |  |
| 235 | M | 0.47 | T | 0.21 |  |  |  | 235 | M | 0.71 |  |  |  |  |
| 236 | A | 0.91 |  |  |  |  |  | 236 | A | 0.99 |  |  |  |  |
| 237 | G | 1.00 |  |  |  |  |  | 237 | G | 1.00 |  |  |  |  |
| 238 | M | 0.90 |  |  |  |  |  | 238 | M | 0.97 |  |  |  |  |
| 239 | A | 0.90 |  |  |  |  |  | 239 | A | 0.97 |  |  |  |  |
| 240 | K | 1.00 |  |  |  |  |  | 240 | K | 1.00 |  |  |  |  |
| 241 | P | 0.97 |  |  |  |  |  | 241 | P | 1.00 |  |  |  |  |
| 242 | L | 1.00 |  |  |  |  |  | 242 | L | 1.00 |  |  |  |  |
| 243 | L | 0.98 |  |  |  |  |  | 243 | L | 0.97 |  |  |  |  |
| 244 | F | 1.00 |  |  |  |  |  | 244 | F | 1.00 |  |  |  |  |
| 245 | H | 1.00 |  |  |  |  |  | 245 | H | 1.00 |  |  |  |  |
| 246 | Q | 0.62 |  |  |  |  |  | 246 | Q | 0.92 |  |  |  |  |
| 247 | K | 1.00 |  |  |  |  |  | 247 | K | 1.00 |  |  |  |  |
| Average |  | 0.93 |  |  |  |  |  |  |  | 0.95 |  |  |  |  |
