# Supplementary material for: Mechanisms for the Evolution of a Derived Function in the Ancestral Glucocorticoid Receptor
Source: PLoS Genet. 2011 Jun 16;7(6):e1002117. doi: 10.1371/journal.pgen.1002117 (PMC3116920; doi:10.1371/journal.pgen.1002117)
Supplement: Table S4 — Data collection and refinement statistics for X-ray crystallography. (DOC) [file pgen.1002117.s006.doc]

| **Data Collection and Refinement Statistics** | |
| --- | --- |
| Resolution (highest shell), Å | 1.95 Å (1.86 – 1.80) |
| Space Group | C2 |
| Unit Cell Dimensions | a=139.4, b=49.1, c=100.4  α=γ=90.0°, β= 108.9° |
| No. of Reflections | 189327 |
| ^a^R_sym_ (highest shell) | 4.8% (%) |
| Completeness (highest shell) | 93.9% (78.3%) |
| Ave. Redundancy (highest shell) | 3.9 (3.3) |
| I/σ | 13.0 (3.6) |
| Monomers per asymmetric unit (AU) | 1 |
| No. of protein atoms/AU | 2518 |
| No. of ligand atoms/AU | 45 |
| No. of waters/AU | 213 |
| ^b^R_working_ (^c^R_free_) | 19.0% (22.33%) |
| *Ave. B*-factors, Å^2^ |  |
| Protein | 28.9 |
| Ligand | 24.7 |
| Water | 40.3 |
| Ion | 53.5 |
| R.m.s. deviations |  |
| Bond lengths, Å | 0.009 |
| Bond angles, ° | 1.1 |
| PDB ID | 3RY9 |
| ^a^ R_sym_ = Σ|I- áIñ|/ Σ|I|, where I is the observed intensity and <I> is the average intensity of several symmetry-related observations.  ^b^ R_working_ = Σ||Fo|-|Fc||/ Σ|Fo|, where Fo and Fc are the observed and calculated structure factors, respectively.  ^c^ R_free_ = Σ||Fo|-|Fc||/ Σ|Fo| for 7% of the data not used at any stage of the structural refinement. | |
