# Supplementary material for: Mechanisms for the Evolution of a Derived Function in the Ancestral Glucocorticoid Receptor
Source: PLoS Genet. 2011 Jun 16;7(6):e1002117. doi: 10.1371/journal.pgen.1002117 (PMC3116920; doi:10.1371/journal.pgen.1002117)
Supplement: Table S5 — FoldX predictions of the change in free energy (ΔΔG) for single and combination mutants of AncCR. Values represent the average of five runs with standard deviation. (DOC) [file pgen.1002117.s007.doc]

| Mutant | ΔΔG (kcal/mol) | SD |
| --- | --- | --- |
| To make AncCR ML |  |  |
| S71C | -0.25 | 0 |
|  |  |  |
| Single Mutants (n = 29) |  |  |
| L1M | 0.06 | 0.37 |
| E11D | -0.26 | 0.04 |
| V13I | -0.13 | 0.09 |
| N19S | 0.18 | 0.14 |
| S20T | 0.46 | 0.00 |
| Q21L | -0.72 | 0.15 |
| K38R | -0.46 | 0.07 |
| V41I | -0.88 | 0.05 |
| V43A | 1.62 | 0.01 |
| I65L | -0.25 | 0.04 |
| C71S | 0.29 | 0.05 |
| K83Q | 0.24 | 0.25 |
| Q88N | 0.67 | 0.02 |
| A107S | -0.28 | 0.02 |
| D110E | -0.41 | 0.20 |
| Q113K | -0.16 | 0.24 |
| R116H | 2.81 | 0.62 |
| Q117K | -0.53 | 0.09 |
| Q120L | -0.58 | 0.05 |
| T128S | -0.91 | 0.03 |
| F132Y | 0.13 | 0.47 |
| S154A | -0.9 | 0.00 |
| M158I | 1.28 | 0.05 |
| N161S | 0.61 | 0.04 |
| R167G | 1.71 | 0.36 |
| R168K | 0.2 | 0.19 |
| Q211E | 0.54 | 0.04 |
| A214T | 0.44 | 0.00 |
| D229N | 0.09 | 0.08 |
|  |  |  |
| Combination Mutants |  |  |
| 43/71 | 1.96 | 0.07 |
| 71/116 | 3.4 | 0.84 |
| 43/116 | 5.31 | 0.27 |
| 43/71/116 | 5.41 | 0.72 |
