# Supplementary material for: Mechanisms for the Evolution of a Derived Function in the Ancestral Glucocorticoid Receptor
Source: PLoS Genet. 2011 Jun 16;7(6):e1002117. doi: 10.1371/journal.pgen.1002117 (PMC3116920; doi:10.1371/journal.pgen.1002117)
Supplement: Supplementary file 7 [file pgen.1002117.s001.doc]

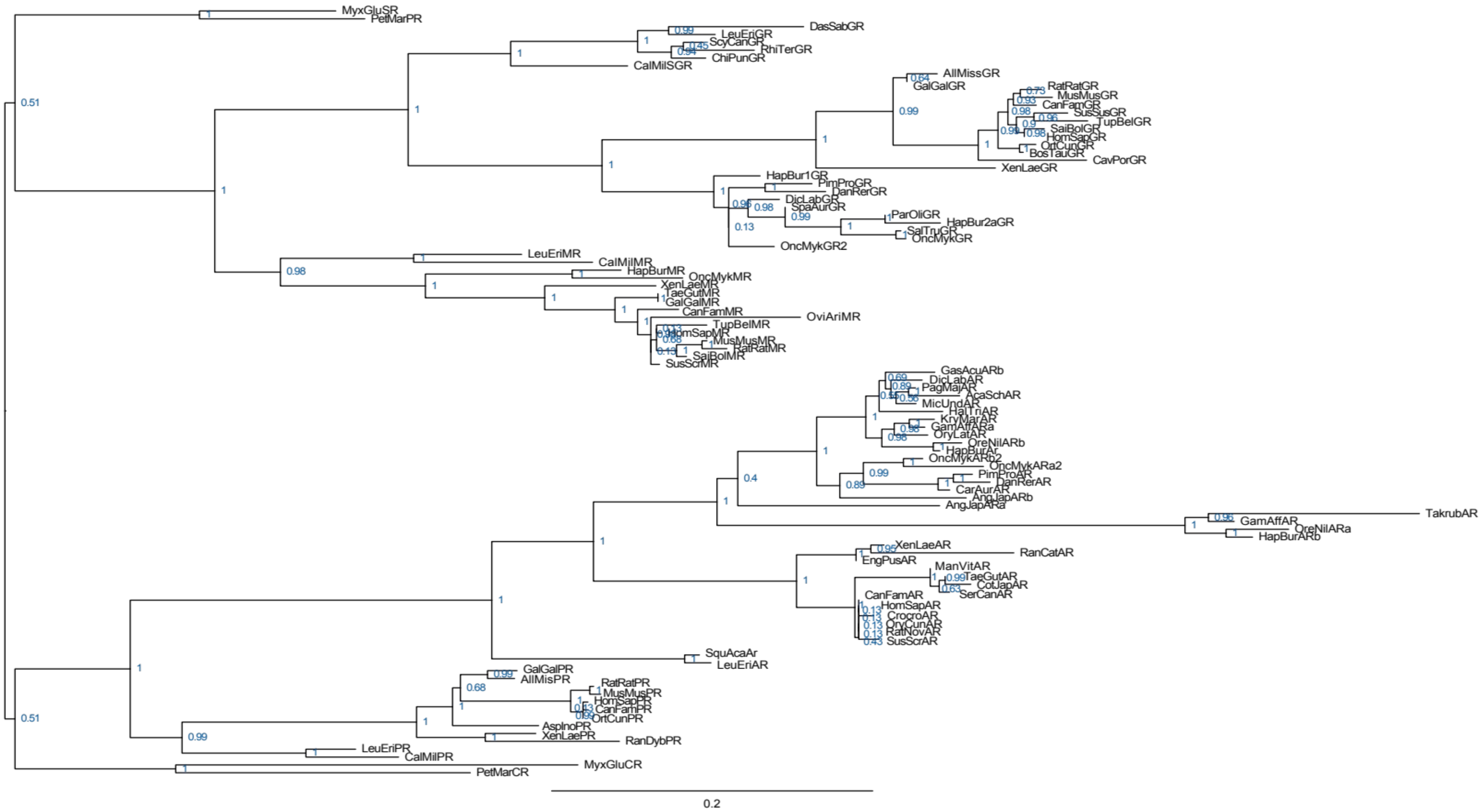
MyxGluSR
PetMarPR
DasSabGR
LeuEriGR
ScyCanGR
RhiTerGR
ChiPunGR
CalMilSGR
AllMissGR
GalGalGR
RatRatGR
MusMusGR
CanFamGR
SusSusGR
TupBelGR
SaiBolGR
HomSapGR
OrtCunGR
BosTauGR
CavPorGR
XenLaeGR
HapBur1GR
PimProGR
DanRerGR
DicLabGR
SpaAurGR
ParOliGR
HapBur2aGR
SalTruGR
OncMykGR
OncMykGR2
LeuEriMR
CalMilMR
HapBurMR
OncMykMR
XenLaeMR
TaeGutMR
GalGalMR
CanFamMR
OviAriMR
TupBelMR
HomSapMR
MusMusMR
RatRatMR
SaiBolMR
SusScrMR
GasAcuARb
DicLabAR
PagMajAR
AcaSchAR
MicUndAR
HalTriAR
KryMarAR
GamAffARa
OryLatAR
OreNilARb
HapBurAr
OncMykARb2
OncMykARa2
PimProAR
DanRerAR
CarAurAR
AngJapARb
AngJapARa
TakrubAR
GamAffAR
OreNilARa
HapBurARb
XenLaeAR
RanCatAR
EngPusAR
ManVitAR
TaeGutAR
CotJapAR
SerCanAR
CanFamAR
HomSapAR
CrocroAR
OryCunAR
RatNovAR
SusScrAR
SquAcaAr
LeuEriAR
GalGalPR
AllMisPR
RatRatPR
MusMusPR
HomSapPR
CanFamPR
OrtCunPR
AspInoPR
XenLaePR
RanDybPR
LeuEriPR
CalMilPR
MyxGluCR
PetMarCR
0.2
